# Switch-like enhancement of epithelial-mesenchymal transition by YAP through feedback regulation of WT1 and Rho-family GTPases

**DOI:** 10.1038/s41467-019-10729-5

**Published:** 2019-06-26

**Authors:** JinSeok Park, Deok-Ho Kim, Sagar R. Shah, Hong-Nam Kim, Kshitiz, Peter Kim, Alfredo Quiñones-Hinojosa, Andre Levchenko

**Affiliations:** 1 Department of Biomedical Engineering and Yale Systems Biology Institute, Yale University, New Haven, CT 06520 USA; 2 Department of Bioengineering and Institute for Stem Cell and Regenerative Medicine, University of Washington, Seattle, WA 98195 USA; 3 Department of Biomedical Engineering, The Johns Hopkins University School of Medicine, Baltimore, MD 21231 USA; 4 Department of Neurologic Surgery, Mayo Clinic, Jacksonville, FL 32224 USA; 5 Center for BioMicrosystems, Korea Institute of Science and Technology, Seoul, 02792 Republic of Korea

**Keywords:** Cell signalling, Epithelial-mesenchymal transition

## Abstract

Collective cell migration occurs in many patho-physiological states, including wound healing and invasive cancer growth. The integrity of the expanding epithelial sheets depends on extracellular cues, including cell-cell and cell-matrix interactions. We show that the nano-scale topography of the extracellular matrix underlying epithelial cell layers can strongly affect the speed and morphology of the fronts of the expanding sheet, triggering partial and complete epithelial-mesenchymal transitions (EMTs). We further demonstrate that this behavior depends on the mechano-sensitivity of the transcription regulator YAP and two new YAP-mediated cross-regulating feedback mechanisms: Wilms Tumor-1-YAP-mediated downregulation of E-cadherin, loosening cell-cell contacts, and YAP-TRIO-Merlin mediated regulation of Rho GTPase family proteins, enhancing cell migration. These YAP-dependent feedback loops result in a switch-like change in the signaling and the expression of EMT-related markers, leading to a robust enhancement in invasive cell spread, which may lead to a worsened clinical outcome in renal and other cancers.

## Abstract (web summary)

Reorganisation of the extracellular matrix (ECM) controls processes involving epithelial-mesenchymal transition (EMT). Here, the authors show that EMT occurring in epithelial cells on a fabricated nano-engineered cell adhesion surface is triggered by mechanical cues from the ECM.

## Introduction

Reorganization of epithelial layers is a major hallmark of developmental process, tissue repair, and pathologies, including tumorigenesis^1–3^. Frequently, during these processes, epithelial organization undergoes transient disintegration and epithelial-to-mesenchymal transition (EMT)^4–6^. Recent studies indicate that the organization of the extracellular matrix (ECM) can dramatically change the collective behavior of epithelial cells and increase the propensity for invasive and metastatic spread of tumors^7–9^, strongly suggesting that ECM organization can be an important regulator of both individual and collective cell migration. Furthermore, ECM reorganization can also control EMT-like processes in various normal developmental settings, e.g., in gastrulation and the onset of neural crest cell migration^10,11^, as well as in would healing^12^. Therefore, it is important to explore how and altered ECM organization may challenge the integrity of the epithelial layers and promote EMT.

A powerful way to explore the effects of ECM organization in cell culture environments is to use structured cell adhesion substrata with arrays of nanoscale aligned fibers similar in size and organization to the naturally occurring aligned ECM fibers. This method has been extensively used to mimic individual cell migration on aligned ECM fibers^13–16^. The movement of cells on such surfaces has displayed many important similarities to migration patterns frequently observed in vivo^17–19^. In the context of aggressive cancer spread, the use of this experimental platform demonstrated not only biomimetic cell migration^20^ but also a striking correspondence with clinical outcomes^21^. This platform can also promote biomimetic collective cell behavior, particularly in the context of large, coupled, and contractile cardiomyocyte tissue-like constructs^22^. The results argue that this adhesion substratum allows one to carefully simulate the presence of the ECM matrix while retaining the convenience and resolution of the common tissue culture technologies.

Combining this experimental approach with normal or cancerous epithelial cell sheets, here we report that the topographic organization of the cell adhesion substratum that mimics aligned ECM fibers can trigger both partial and full EMT-like processes. Furthermore, we provide evidence that this phenomenon depends on an all-or-none, switch-like activation of YAP (Yes-associated protein), a transcriptional co-regulator previously implicated in the control of cell–cell and cell–substratum interactions, and in mechanosensing^23–26^. We delineate the molecular mechanisms involved in this switch-like response, demonstrating that it depends on feedback interactions involving Wilms tumor-1 (WT1)-dependent crosstalk of YAP with E-cadherin, and Merlin-dependent crosstalk of YAP with Rho-family small GTPases, uncovering previously unknown regulatory effects of YAP. The switch-like downregulation of E-cadherin and upregulation of cell speed can lead to partial EMT in large swathes of submarginal cells in advancing sheets, and full EMT at the edges of the sheets. Overall, our results strongly argue that YAP-mediated, switch-like responses to changes in ECM organization can be a key factor determining the integrity of epithelial sheets and the onset of EMT.

## Results

### Aligned ECM topography can trigger the EMT-like phenotype

**Fig. 1:**
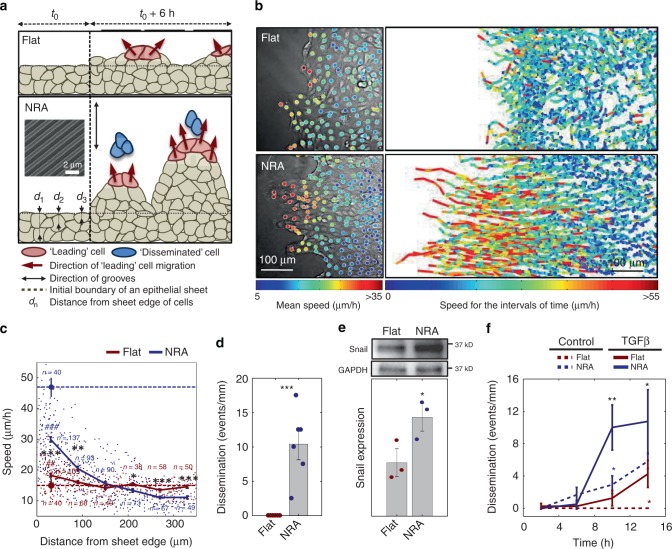
Anisotropic texture of the mechanical environment increases EMT. **a** Schematic depiction of the edge areas of the expanding epithelial sheets, including formation of the finger-like protrusions (FLPs) led by “leading” cells and occasional separation of the tip cells (“dissemination”). It also defines the distance metric for the cell migration analysis in this and subsequent figures. The inset shows the electron micrograph of the NRA. **b** Cell trajectories colored to reflect the cell speed values in an expanding sheet on flat surfaces (top) and NRA (bottom). The colors of each dot indicate the mean speed values of the corresponding cells (left). The trajectories were tracked for 6 h with the speed measured every 20 min (right). **c** Migration speed of individual cells on flat and NRA substrata at different initial distances from the edge of the sheet (corresponding to values of *d* in panel **a**). Each dot represents the average speed of an individual cell. Dashed lines indicate the averaged speed of isolated individual cells on a flat surface (red) and NRA (blue) (each number of independently analyzed cells, *n*, is indicated, # = statistical significance of speed on the marginal region vs. the most submarginal region of cells on a flat surface (red) and NRA (blue), ^##^*P* < 5 × 10^−4^ and ^###^*P* < 5 × 10^−6^, * = statistical significance of the speed of cells on a flat surface vs. NRA, and *P < 0.05, **P < 5 × 10^−4^, and ***P < 5 × 10^−6^). **d** Disseminations from epithelial sheets per unit length of epithelial sheets on flat substrata and NRA for 18 h (*n* = 4 biologically independent experiments, * = statistical significance of numbers of dissemination on flat surfaces vs. NRA. ***P < 5 × 10^−6^). **e** The expression of Snail in the cells on flat surfaces and NRA analyzed by immunoblotting (*n* = 3 biologically independent samples, * = statistical significance of Snail expression in cells on flat vs. NRA substrata, *P < 0.05). **f** Dissemination from epithelial sheets on flat substrata and NRA in the presence of TGFβ (*n* = 4 biologically independent samples, * = statistical significance of the number of disseminations with control vs. TGFβ on the flat substratum (red) and on NRA (blue), flat substrata vs. NRA with TGFβ (black), *P < 0.05 and **P < 1 × 10^−2^). All error bars are S.E.M and statistical significance was determined by two-sided Student’s *t*-test

We explored the putative mechanistic underpinnings of the effects of the nanotopographic ECM structure on epithelial sheet expansion by culturing cells on arrays of nanoscale aligned ECM fiber-like ridges coated with collagen type I. The feature size of topographic structures approximates the diameter of ECM fibers, in the submicron range, known to induce profound changes in single-cell and collective responses of various cells types^16,27–29^. Prior to analysis, epithelial sheets of Madin–Darby canine kidney (MDCK) cell sheets were formed inside a constraining stencil, which was then lifted to allow sheet expansion, on the structured substrata and flat controls. Consistent with prior observations^30^, on these nanostructured surfaces termed henceforth the nanostructured ridge arrays (NRA), cells displayed a 50% increase in the migration velocity with enhanced persistence of cell migration in the direction of the fiber-like ridges vs. the control flat surfaces (Fig. 1a–c, Supplementary Fig. 1 and Supplementary Movie 1). The cell migration speed on NRA was maximal at the edges of expanding sheets, gradually decreasing deeper into the sheet vs. much more even-speed distribution on the flat surfaces (Fig. 1c and Supplementary Fig. 1). On NRA, the edges of the sheets, although initially straight, displayed a dramatic increase in the formation of finger-like projections (FLPs), with the cells at the tips of the projections displaying a distinct cytoskeleton structure (Supplementary Figs. 2 and 3, and Supplementary Movies 2 and 3) and increased polarization (Supplementary Fig. 4), in contrast to less pronounced effects on the flat surfaces (cf. with ref. ^31^). Epithelial cells also frequently separated from the tips of the FLPs and moved away with an enhanced speed, similar to the speed of single cells plated on the same surface, in a behavior resembling full EMT (Fig. 1d, Supplementary Fig. 5, and Supplementary Movie 4).

At the molecular level, EMT is frequently characterized by expression and localization of well-established markers, such as β-catenin, Snail, Slug, Twist, and vimentin^4^. Indeed, we found higher Snail and vimentin expressions in cells cultured on NRA vs. flat surfaces (Fig. 1e and Supplementary Fig. 6a) and increased localization of Slug, Twist, and vimentin in FLPs at the edge of the cell sheet on NRA but not flat surfaces (Supplementary Fig. 6b–d). These results further supported EMT-like nature of the epithelial cell expansion on NRA, particularly at the FLP structures at the edges of the expanding sheets.

To test whether the substratum nanotopography effects are synergistic with more established EMT inducers, such as TGFβ^32^, we examined the influence of TGFβ stimulation on epithelial sheet expansion on surfaces with distinct topographies. We found that, although, as expected, the number of cell dissemination events was greatly increased in the presence of TGFβ on the flat surfaces, the effect was similar to the effect of NRA. Strikingly, when TGFβ and NRA cues were combined, the number of dissemination events increased fourfold vs. the effect of each of these cues alone (Fig. 1f), indicating a strong synergy between these two inputs. Overall, these data argued that an EMT-like process could be induced by nanotopographic cues in a fashion distinct from but synergistic with TGFβ.

### Switch-like regulation of YAP by mechanical cues mediates EMT

**Fig. 2:**
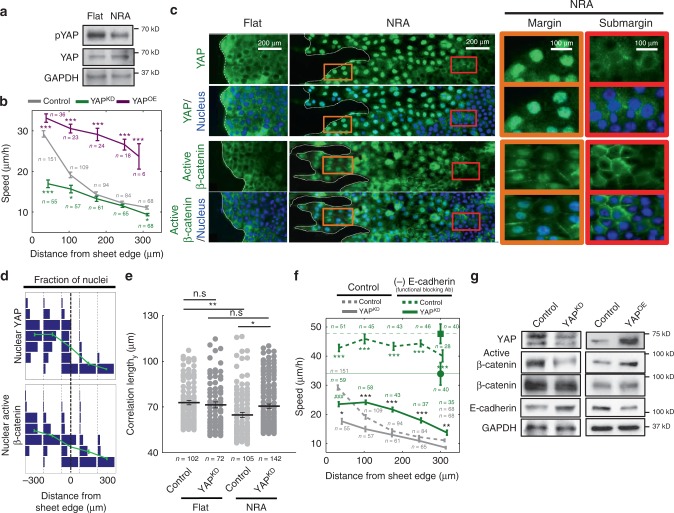
Switch-like regulation of YAP in expanding epithelial layers loosening cell–cell adhesion. **a** Cells on flat substrata and NRA analyzed by immunoblotting using YAP and phosphorylated YAP antibodies. **b** Cell migration speed of individual YAP^KD^ and YAP^OE^ cells in cell sheets as a function of the distance from the sheet edge on NRA (each number of independently analyzed cells, *n*, is indicated, * = statistical significance of speed of control vs. YAP^KD^ cells on NRA (green) and of control vs. YAP^OE^ cells on NRA (purple), *P < 0.05, **P < 5 × 10^−4^, and ***P < 5 × 10^−6^). **c** Immunofluorescence staining for YAP and active β-catenin in epithelial cell sheets on flat substrata and NRA. Translocation of YAP and active β-catenin into nuclei observed in marginal zones and FLPs of sheets expanding on NRA (brown boxes) and mostly cytoplasmic YAP and active β-catenin localization in submarginal cells on NRA (red boxes) and on flat substrata. The samples were analyzed after 8 h from the initiation of sheet expansion. **d** Fractions of nuclei displaying different intensities of YAP and active β-catenin staining as a function of the distance from the sheet edge determined at 8 h after initiation of sheet expansion (the edge is coincident with the concave regions at the bases of FLPs in panel **c**). **e** Velocity correlation length in y-direction, parallel to the direction of epithelial expansion for the control and YAP^KD^ cells on flat substrata and NRA (each number of independently analyzed cells, *n*, is indicated, * = statistical significance of correlation length, *P < 0.05, **P < 0.01, and n.s = no significance). **f** Cell migration speed of individual cells in cell sheets as a function of the distance from the sheet edge on flat substrata and NRA, in the presence of an E-cadherin functional blocking antibody. Dashed line with a square marker indicates the average cell migration speed of isolated control cells and solid line with a circle marker corresponds to that of isolated YAP^KD^ cells (each number of independently analyzed cells, n, is indicated, # = statistical significance of speed values in the marginal region vs. the most submarginal region of YAP^KD^ cells with drugs, ^###^*P* < 5 × 10^−6^. * = statistical significance of control vs. YAP^KD^ cells with drugs (green) and of YAP^KD^ cells with vs. without drugs (black), *P < 0.05, **P < 5 × 10^−4^, and ***P < 5 × 10^−6^). **g** Control, YAP^KD^, and YAP^OE^ cells were immunoblotted using active β-catenin and E-cadherin antibodies. All error bars are S.E.M and statistical significance was determined by two-sided Student’s *t*-test

What is the mechanism of the dramatic increase in the EMT-like events at the edges of epithelial sheets expanding on NRA? We focused on the analysis of YAP (Yes-associated protein 1), since this transcriptional regulator has been implicated both as a mechanosensor^24^ and as a regulator of the balance between cell–cell^13^ and cell–ECM interactions^23^. YAP is also known to promote wound healing^33^ and certain EMT-like characteristics^34^. Indeed, we found that cells on NRA surfaces exhibited decreased phosphorylation of YAP, indicating its enhanced activity, compared with cells cultured on flat substrata (Fig. 2a). Disrupting the expression of YAP (and its TAZ) by RNA interference substantially reduced the cell migration speed (Fig. 2b, Supplementary Figs. 7 and 8, and Supplementary Movie 5) and the extent of FLP formation, essentially to the levels seen in the unperturbed cells on flat substrata. In contrast, YAP overexpression promoted cell migration, suggesting that YAP activity indeed mediated the mechanosensing-dependent aggressive epithelial monolayer expansion (Fig. 2b).

When examined at the single-cell level, YAP localization was strikingly distinct in cells cultured on different substrata. Whereas it was primarily cytosolic in the cells cultured on flat substrata, it displayed an all-or-none switch in nuclear/cytosolic distribution on NRA surfaces—suddenly changing from nuclear in cells proximal at the sheet edge (in a region extending multiple cell diameters into the sheet) to cytosolic in the bulk of the monolayer (Fig. 2c). This finding could be captured by a bimodal distribution of nuclear YAP across the epithelial cell population (Fig. 2d). A change of the substratum coating to 20 μg/ml of fibronectin (FN) led to an enhanced nuclear YAP translocation even on the flat substratum (albeit only in marginal cells) (Supplementary Fig. 9), but again, we found that the localization was strongly enhanced on NRA, in a switch-like fashion. This result suggested that although YAP was responsive to both chemical and mechanical inputs, the strong and switch-like effect of NRA on nuclear translocation of YAP was primarily mechanical in nature.

To complement the analysis of YAP localization, we examined the intracellular distribution of active β-catenin, another EMT marker displaying various regulatory interactions with YAP^35,36^. We again observed its distinct, switch-like localization patterns on NRA vs. flat substrata. Specifically, we found that active β-catenin was entirely plasma membrane associated in cells on the flat substrata, whereas it abruptly switched from mostly nuclear in areas proximal to sheet edges to mostly plasma membrane localized in the cells in the bulk of the sheet, with the switch again well described by a bimodal distribution of nuclear localization of active β-catenin (Fig. 2c, d). Furthermore, YAP knockdown led to a reversal of localization of active β-catenin to the plasma membrane in virtually all cells (Supplementary Fig. 10a). Staining for vimentin revealed a substantial reduction of its expression in YAP^KD^ cells vs. unperturbed cells (Supplementary Fig. 10b), whereas *CDH1* (E-cadherin) mRNA levels increased and *Snai1* (Snail) mRNA levels decreased in YAP^KD^ cells (Supplementary Fig. 10c). These results strongly suggested a critical role for YAP in inducing EMT markers in cell layers adjacent to the moving front of epithelial sheets on aligned fibrous cell adhesion substrata.

### YAP induces EMT through feedback from E-cadherin via WT1

We next explored the mechanisms of the switch-like YAP activation. We first explored how YAP might control the expression of E-cadherin (Supplementary Fig. 10c). We found a lower level of *CDH1* mRNA expression on NRA, consistent with YAP upregulation on this substratum (Supplementary Fig. 11a). The correlation length of cell velocities, which is a functional metric of collective cell migration due to cell coupling through cell–cell adhesion^37^, was significantly decreased on NRA vs. flat surfaces, consistent with lower E-cadherin-mediated cell–cell adhesion (Fig. 2e). Furthermore, the correlation of cell migration on NRA was fully restored in YAP^KD^ cells, again underscoring the critical role of YAP in E-cadherin-mediated cell–cell coupling (Fig. 2e), consistent with its effect on cell dissemination (Supplementary Fig. 7). We further found that inhibition of E-cadherin-mediated cell–cell interaction by an E-cadherin blocking antibody, which led to a profound increase in cell dissemination, was partially rescued by the YAP knockdown (Fig. 2f and Supplementary Movie 6). These data suggested that YAP has a negative effect on E-cadherin function. Consistent with this functional effect, on the biochemical level, we also observed not only a substantial increase in E-cadherin protein levels and suppression of β-catenin activity in YAP^KD^ cells, consistent with the increased *CDH1* expression observed before, but we also found a decrease in E-cadherin expression and increase in β-catenin activation in cells overexpressing YAP (YAP^OE^) (Fig. 2g). Overall, these results suggested that YAP can control E-cadherin expression and function in epithelial cells, raising the question of the mechanisms of this regulation.

**Fig. 3:**
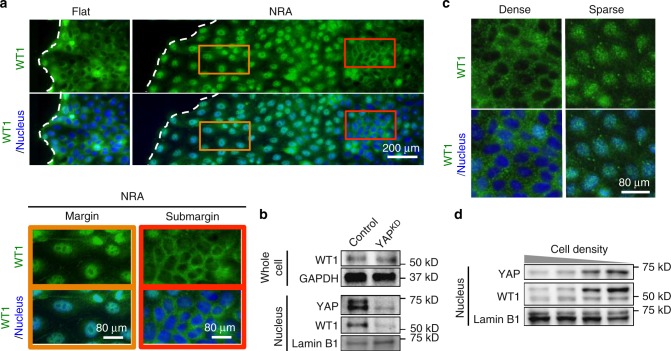
Similar subcellular localization of WT1 with YAP. **a** Immunofluorescence staining for WT1 in epithelial cell sheets on flat substrata and NRA. Translocation of WT1 into nuclei was observed in marginal zones and FLPs of sheets expanding on NRA (brown boxes). Submarginal cells on NRA (red boxes) and on flat substrata showed YAP in the cytoplasm. The samples were fixed after 8 h to remove stencils. **b** Control and YAP^KD^ cells and their nuclear fractions were analyzed using immunoblotting using YAP and WT1 antibodies. **c** Immunofluorescence staining for WT1 in cells and **d** immunoblotting of YAP and WT1 abundance in the nuclei in different density–epithelial cell sheets showing density-dependent translocation of WT1 into the nucleus

To further explore the mechanistic details of the putative E-cadherin regulation by YAP, we examined the known suppressor of E-cadherin expression, the Wilms tumor protein (WT1)^38,39^. This protein is particularly interesting to evaluate, due to its role in regulating mesenchymal–epithelial transition (MET), and cell–cell interactions in the developing kidney (making MDCK cells a relevant cell-type model) and the associated malignancies^40^. Surprisingly, we found that WT1 localization was very similar to the nuclear and cytoplasmic YAP localization patterns across the expanding epithelial layer (Fig. 3a). Furthermore, silencing of YAP expression led to a decrease in the nuclear localization of WT1 (Fig. 3b). Moreover, we found that WT1 and YAP displayed a correlated decrease of nuclear localization with increasing cell density (Fig. 3c, d). Importantly, the expression of WT1, as quantified by immunoblotting, did not display a difference between cells cultured on flat surfaces and NRA (Supplementary Fig. 11b), suggesting that any putative effects of WT1 on the YAP-mediated EMT phenotype would depend on post-translational regulation. The localization patterns suggested that this post-transcriptional regulation might occur through a physical interaction with and thus intracellular trafficking of WT1 with YAP. This hypothesis was explored in the following experiments.

**Fig. 4:**
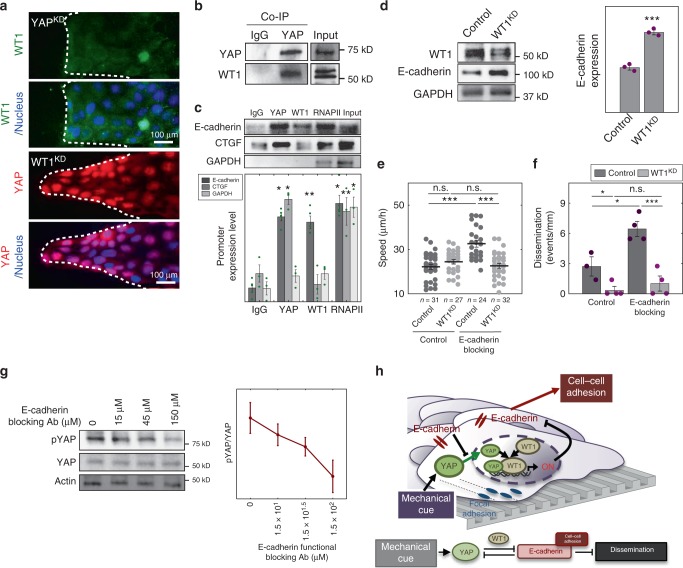
YAP regulates E-cadherin through WT1 in epithelial layers on NRA. **a** Immunofluorescence staining for WT1 in YAP^KD^ cell sheets (top), and for YAP in WT1^KD^ cell sheets cultured on NRA. The samples were fixed after 8 h to remove stencils. **b** Co-IP analysis using the YAP antibody, followed by immunoblotting using the WT1 antibody. **c** Chromatin immunoprecipitation (ChIP) analysis of the WT1-YAP complex binding at the E-cadherin promoter (*n* = 3). We extracted cross-linked chromatin and immunoprecipitated it using antibodies against YAP, WT1, IgG (negative control), and RNAPII or histone H3 (see Supplementary Fig. 12a) antibodies (positive controls). The immunoprecipitated chromatin and input genomic DNA were used for amplification of the E-cadherin promoter, CTGF promoter (known to be regulated by YAP but not WT1, and used as another control), and GAPDH promoter (*n* = 4 biologically independent samples, * = statistical significance of PCR products from each sample vs. IgG, *P < 0.05 and **P < 0.01). **d** E-cadherin expressions of control and WT1^KD^ cells analyzed by immunoblotting with WT1 and E-cadherin antibodies (*n* = 3 biologically independent samples, * = statistical significance of E-cadherin expression of control and WT1^KD^ cells, ***P < 5 × 10^−3^). **e** Cell migration speed of individual cells in control and WT1^KD^ epithelial cells in the marginal region of the sheets in the presence of an E-cadherin blocking antibody (each number of independently analyzed cells, *n*, is indicated, * = statistical significance, n.s = no significance, and ***P < 5 × 10^−3^). **f** Dissemination of cells in control and WT1^KD^ epithelial sheets on NRA in the presence of an E-cadherin blocking antibody (*n* = 4 biologically independent experiments, * = statistical significance, *P < 0.05, ***P < 5 × 10^−3^, and n.s = no significance). **g** Immunoblotting of phosphorylated YAP and total YAP in cell sheets cultured in the presence of different concentrations of E-cadherin blocking antibody (*n* = 3 biologically independent samples). **h** Schematic of the YAP-mediated cell dissemination triggered by mechanical cues stemming from NRA, operating through E-cadherin control by YAP-WT1 complexes and leading to cell dissemination in epithelial cell sheets on NRA. All error bars are S.E.M and statistical significance was determined by two-sided Student’s *t*-test

First, we further analyzed codependence of YAP and WT1 localization patterns. We observed that YAP^KD^ cells showed no nuclear WT1 localization on NRA, suggesting that YAP is necessary to facilitate transport of WT1 into the nucleus (Fig. 4a). Conversely, WT1^KD^ cells under the same conditions still displayed nuclear YAP, suggesting that YAP localization patterns are not affected by WT1, and thus YAP can be the vehicle enhancing nuclear WT1 localization, but not vice versa. We then explored if YAP and WT1 formed a physical complex. Indeed, our co-immunoprecipitation experiments indicated WT1-YAP complex formation, raising the possibility that these two transcriptional regulators can be jointly trafficked and control gene expression (Fig. 4b). This result putatively linked YAP to regulation of WT1-dependent genes, including E-cadherin. To explore whether the WT1-YAP complex directly controls E- cadherin expression, we performed chromatin immunoprecipitation with YAP and WT1 antibodies at E-cadherin promoter sequence^41^ and expression analysis using E-cadherin (*CDH1*) promoter-coupled luciferase reporter^42^ (Fig. 4c and Supplementary Fig. 12). This analysis confirmed that the WT1-YAP complex binds directly to the E-cadherin promoter sequence, controlling *CDH1* gene expression (Fig. 4c). Furthermore, we found that WT1 knockdown resulted in an increased E-cadherin mRNA transcription and protein expression in MDCK cells, supporting a negative effect of WT1 on E-cadherin expression (Fig. 4d and Supplementary Fig. 13a). These results were buttressed by the finding that silencing of YAP expression led to an increase of E-cadherin mRNA in cells on NRA (Supplementary Figs. 10c and 13a).

Our initial findings suggested that YAP can control both cell migration speed and E-cadherin-mediated cell–cell coupling. However, it was not clear if either or both of these two effects occurred through regulation of WT1. Hence, we examined the effects of WT1 silencing on both speed and E-cadherin-dependent cell coupling. We found that WT1 knockdown did not have a significant effect on cell migration speed in epithelial cell sheets cultured on collagen-coated NRA (Fig. 4e), suggesting that WT1 may mediate the YAP-mediated decrease in cell–cell coupling but not the effect of YAP on migration speed. Indeed, WT1 silencing significantly reduced cell dissemination, underscoring its role in controlling E-cadherin-mediated cell adhesion, and its enhancement by NRA (Fig. 4f). We then explored the effect of WT1 on both cell migration and dissemination in cells with decreased E-cadherin-mediated coupling, using E-cadherin blocking antibody. As observed above, decoupling of E-cadherin-mediated cell interactions led to an increased cell dissemination, and thus, indirectly, higher average speed (Fig. 4e, f, and Supplementary Movie 7). As expected, WT1 silencing led to the rescue of both these phenotypes, further suggesting that E-cadherin regulation is the mode by which WT1 regulates the EMT-like phenotype on NRA (Fig. 4e, f). The results were consistent with the effects of WT1 perturbations in cells cultured on FN-coated NRAs (although the indirect effect on cell speed was more significant in this case) (Supplementary Fig. 13b). In combination, these results suggested that WT1 controls EMT triggered by NRA by affecting E-cadherin-mediated cell coupling rather than the cell migration. Below, we explore how YAP can control cell migration in a WT1-independent fashion.

Our results suggest that YAP, through interaction with WT1, can downregulate E-cadherin expression. Previously, E-cadherin has been reported to downregulate YAP^43^. We indeed found that E-cadherin blocking antibody could upregulate YAP in MDCK cells (Fig. 4g). These results suggested a double-negative feedback, with E-cadherin and YAP mutually inhibiting each other (Fig. 4h). Such feedback interactions can create switch-like changes in protein expression and activity, as demonstrated in a mathematical model below, leading to a switch-like onset of YAP and WT1 nuclear localization in the vicinity of the advancing margins and account for the E-cadherin-dependent EMT-like phenomena.

### YAP controls cell migration speed through feedback from Rac1

**Fig. 5:**
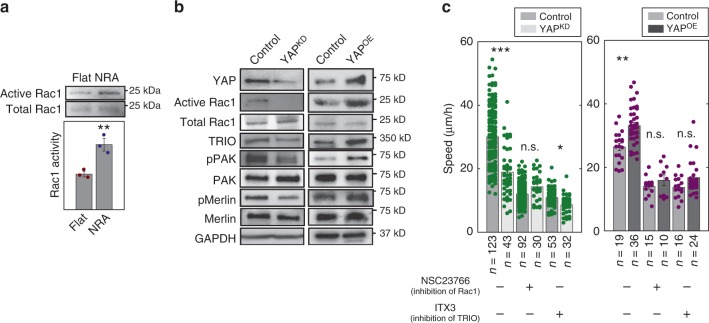
Cell migration-enhancing effect of YAP via Rac1. **a** Rac1 activity assay using cells cultured on flat surfaces and NRA (*n* = 3 biologically independent samples, * = statistical significance of the ratio of active Rac1 to Rac1 on flat surfaces vs. NRA. *P < 0.05). **b** Control, YAP^KD^, and YAP^OE^ cells were immunoblotted to evaluate Rac1 activity via pull-down assay, expression of TRIO, and phosphorylation of PAK and Merlin. **c** Cell migration speed of individual cells in YAP^KD^ (left) and YAP^OE^ (right) cells in the marginal region of epithelial cell sheets on NRA in the presence of Rac1 inhibitor, NSC23766 and a TRIO inhibitor, ITX3 (each number of independently analyzed cells, n, is indicated, * = statistical significance of control vs. YAP^KD^ and control vs. YAP^OE^ cells with drugs, n.s = no significance, *P < 0.05, **P < 1 × 10^−2^, and ***P < 5 × 10^−3^). All error bars are S.E.M and statistical significance was determined by two-sided Student’s t-test

Our results so far implicated YAP in the control of E-cadherin-mediated cell coupling and EMT-like cell dissemination. However, it is not clear how YAP might also control the speed of cell migration. To investigate this effect, we focused on the key small Rho-family GTPase controlling cell polarity and migration, Rac1^44–46^. We found that cells cultured on NRA displayed enhanced activity of Rac1 vs. flat surfaces, suggesting that YAP can control the activity of this small GTPase (Fig. 5a). Indeed, YAP silencing abolished Rac1 activation, and conversely, increasing YAP expression led to an increased Rac1 activation (Fig. 5b). Interestingly, YAP knockdown also downregulated the expression of TRIO, a Rac1-activating GTP-exchange factor, and downregulated the phosphorylation of a Rac1 effector, p21-activated kinase (PAK), whereas YAP overexpression had the opposite effects, further suggesting that YAP-mediated Rac1 regulation is functionally significant and dependent on TRIO (Fig. 5b). Indeed, there is TRIO phenocopied inhibition of the Rac1 activity in the cell migration assay (i.e., it led to suppression of enhanced motility), suggesting that YAP can control Rac1 activation and cell migration, at least in part, through regulation of TRIO expression (Fig. 5c, Supplementary Fig. 14, and Supplementary Movies 8 and 9; also see Sagar et al.^47^ demonstrating transcriptional TRIO regulation by YAP in other cell types).

**Fig. 6:**
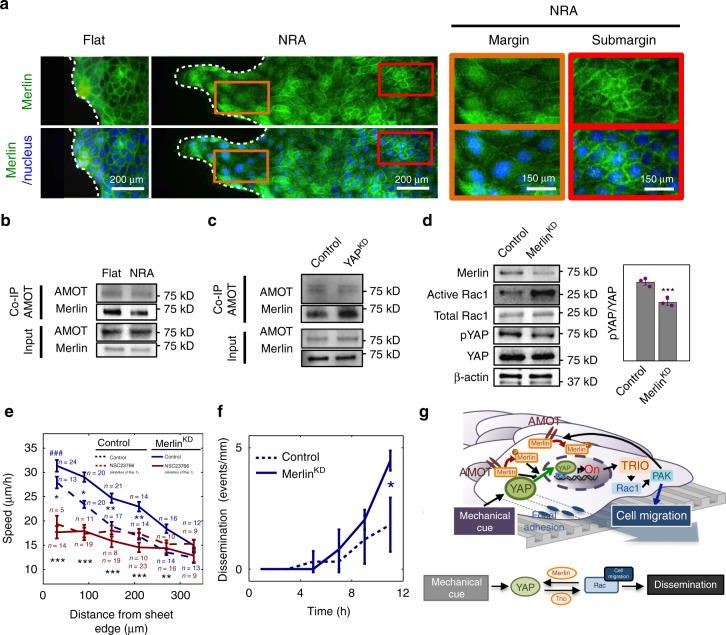
Cross-regulation of YAP, Rac1, and Merlin in epithelial layers on NRA. **a** Immunofluorescence staining for Merlin in epithelial cell sheets on a flat surface and NRA. In the marginal zone and FLPs of sheets on NRA, Merlin was primarily localized in the cytosol (brown boxes), whereas it was localized in the cell–cell contacts of submarginal cells cultured on NRA and flat substrata (red boxes). The samples were fixed after 8 h to remove stencils. **b** Cells on flat surfaces and NRA were used for Co-IP analysis with the AMOT antibody for investigating physical bounding between AMOT and a target protein, Merlin, and the precipitates were immunoblotted using Merlin antibody. **c** Lysates from control and YAP^KD^ cells were used for co-immunoprecipitation (Co-IP) study using the AMOT antibody, analyzed by immunoblotting with Merlin antibody. **d** Control and Merlin^KD^ cells were immunoblotted for evaluation of Rac1 activity via pull-down assay, YAP expression, and its phosphorylation. (*n* = 3 biologically independent samples, * = statistical significance of the ratio of active Rac1 to Rac1 on control vs. Merlin^KD^ cells. ***P < 5 × 10^−3^). **e** Cell migration speed of individual cells in Merlin^KD^ epithelial cell sheets as a function of the distance from the sheet edge in cell sheets cultured on NRA in the presence of a Rac1 inhibitor, NSC23766. (each number of independently analyzed cells, *n*, is indicated, # = statistical significance of speed values in the marginal region vs. the most submarginal region of Merlin^KD^ cells, ^###^*P* < 5 × 10^−6^. * = statistical significance of control vs. Merlin^KD^ cells without drugs (blue) and of Merlin^KD^ cells with vs. without drugs (black), *P < 0.05 and **P < 5 × 10^−3^). **f** Dissemination of cells in control and Merlin^KD^ epithelial sheets on NRA. All error bars are S.E.M. (*n* = 4 biologically independent experiments, * = statistical significance of the number of disseminations of cells from control vs. Merlin^KD^ epithelial sheets, *P < 0.05). **g** Schematic of YAP-mediated signaling cascades operating via Rac1 and Merlin, controlling cell speed in cells undergoing partial and complete EMT. All error bars are S.E.M and statistical significance was determined by two-sided Student’s *t*-test

We then explored the possibility that Rac1 can in turn control YAP activity in cells displaying enhanced motility, thus forming another feedback-regulatory loop. One candidate for this putative feedback regulation is Merlin, a negative regulator of YAP^48^ also known to control and be controlled by Rac1 and PAK activity^49^. In agreement with the feedback hypothesis, we found that silencing YAP expression resulted in a decreased Merlin phosphorylation (Fig. 5b), consistent with prior reports indicating that enhanced YAP activity upregulates Merlin^49,50^. This feedback, involving cross-regulation of Rac1, PAK, Merlin, and YAP may be enhanced in epithelial cells on NRAs. This was in agreement with the finding that culturing cells on NRA (but not flat surfaces) decreased the association of Merlin with cell–cell junctions, particularly in FLPs (Fig. 6a), consistent with reduced association of Merlin with angiomotin (AMOT), a plasma membrane-bound protein localized to cell–cell junctions (Fig. 6b). The biochemical interaction between YAP and Merlin was further supported by downregulation of Merlin phosphorylation in YAP^KD^ cells and upregulation of Merlin phosphorylation in YAP^OE^ cells (Fig. 5b), as well as increased association of Merlin with AMOT in YAP^KD^ cells (Fig. 6c). This result was also consistent with the prior findings that a decrease in phosphorylation suppresses Merlin activity through controlling its association with AMOT^37,51,52^. Importantly, Merlin silencing both increased Rac1 activity and decreased the phosphorylation (and thus increased the activity) of YAP (Fig. 6d), leading to a significant increase in cell migration speed, which was reversed by Rac1 inhibition (Fig. 6e and Supplementary Movie 10). Merlin knockdown also led to a significantly greater occurrence of cell dissemination events (Fig. 6f). In combination, these diverse results supported the feedback circuit, in which Merlin can negatively control YAP, which in turn negatively controls Merlin, in a Rac1- and PAK-dependent fashion, in cells cultured on NRA (Fig. 6g). This circuit, involving a positive cross-regulation between YAP and Rac1, can result in a switch-like increase in cell migration, following a sufficiently strong activation of YAP by mechanical cues, such as the fibrous substrata studied here.

Successful cell migration depends on a balance of activities of Rac1 and another small GTPase of the Rho family, RhoA. These two small GTPases display mutually inhibitory cross-regulation thought to be critical for cell polarization and optimal cell migration^46,53^. We hypothesized that, if YAP enhances the activity of Rac1, it might therefore also indirectly suppress the activity of RhoA, and, as a downstream effect, the myosin activation through phosphorylation of myosin light chain (MLC). We indeed found an increase in MLC phosphorylation following silencing of YAP expression, which was particularly enhanced at the cell sheet edges (Supplementary Fig. 15a), supporting a putative effect of YAP on RhoA^54,55^. Indeed, we found that YAP knockdown led to enhanced MLC phosphorylation (Supplementary Fig. 15b), but, in contrast to a recent report^23^, it did not affect the overall MLC expression level. We also observed that suppression of cell migration due to YAP knockdown was rescued by inhibition of the activity of Rho-dependent kinase (ROCK) in cells cultured on NRA (Supplementary Fig. 15c and Supplementary Movie 11). Interestingly, in the absence of YAP silencing, the effect of ROCK inhibition on the migration speed in cells on NRA was limited (Supplementary Fig. 15c), suggesting that NRA-induced YAP activity can suppress ROCK very effectively, underscoring the YAP-dependent role of this cue. Overall, these results suggested that the aligned topography of the ECM-like environment can strongly enhance cell motility through a feedback interaction involving a positive cross-regulation between YAP and Rac1 (and indirect downregulation of RhoA), mediated by Merlin, TRIO, and other components of the signaling networks identified above.

### Feedback involving YAP promotes switch-like onset of EMT

**Fig. 7:**
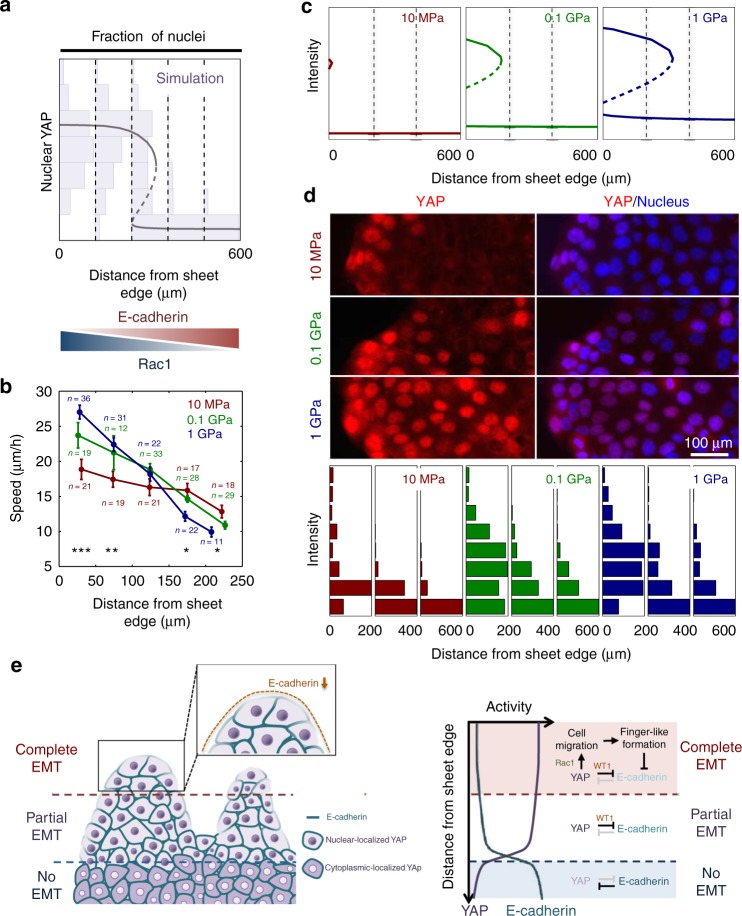
Double-feedback loop with YAP activated on NRA regulating EMT. **a** Simulation result showing that feedback mechanisms lead to emergence of two stable states of high and low YAP activity, indicating epithelial (low Rac1, high E-cadherin, and low YAP) and partial EMT (high Rac1, low E-cadherin, and high YAP) states depending on Rac1 and E-cadherin activity in this system. **b** Cell migration speed on NRAs with different rigidity values, suggesting that rigidity can regulate the basal rate of YAP activation at different initial distances from the edge of the sheet (all error bars are S.E.M, each number of independently analyzed cells, *n*, is indicated, * = statistical significance of the speed of cells on NRA in which the rigidities are 10 MPa vs. 1 GPa, *P < 5 × 10^−2^, **P < 1 × 10^−2^, and ***P < 5 × 10^−3^, all two-sided Student’s *t*-test). **c** Simulated bimodal distribution of YAP activity as a function of the distance from the sheet edge on NRA substrata of different rigidity values. **d** Rigidity-dependent YAP localization in nuclei of cells cultured on NRA. Immunofluorescence staining of YAP showing nuclear localization at different distances from the sheet edge on NRAs with different rigidity values (top). Fractions of nuclei displaying different intensities of YAP staining as a function of the distance from the sheet edge on NRA having different rigidity (bottom) (see details in Supplementary discussion and Supplementary Fig. 17). The samples were fixed after 6 h to remove stencils and induce epithelial expansion. **e** Schematic description of the regulation of EMT by YAP-mediated topographically induced mechanical input, showing full EMT in marginal cells exposed to cell-free areas at the fronts of FLPs, partial EMT in extensive marginal areas, and epithelial organization (no EMT) in the areas most distant from the edge

The switch-like, bimodal nuclear translocation of YAP, WT1, and β-catenin (Figs. 2c and 3a) in the large marginal and submarginal zones of expanding sheets is suggestive of the underlying bistability in regulation of these proteins, mediated by a positive and double-negative-feedback interactions^54,56,57^. Indeed, our data suggest that YAP can negatively control E-cadherin expression through interaction with WT1 (Fig. 2g and ref. ^43^), and be negatively controlled by E-cadherin (Fig. 4g), constituting a double-negative feedback between YAP and E-cadherin. In addition, our results reveal that YAP can be a part of a positive feedback involving downregulation of Merlin and upregulation of Rac1 (Fig. 6g). When incorporated into a mathematical model, this combination of positive- (YAP and Rac1) and double-negative- (YAP and E-cadherin) feedback interactions triggered or modulated by mechanical cues indeed predicted a switch-like enhancement of the EMT phenotype (Fig. 7a and Supplementary Discussion). This model with the corresponding parameters (Supplementary Tables 1 and 2) provided an excellent fit to the bimodal distributions of nuclear localization of YAP at different distances from the sheet edge, placing maximum bimodality at the bases of forming FLPs, and providing a mechanism for instability at the sheet fronts, leading to FLP formation. To validate this model, we perturbed the system by additionally varying the mechanical cues stimulating YAP activity. We achieved this by further modifying NRA substrata using materials with different effective stiffness. YAP is known to be responsive to this type of mechanical cue^24,58,59^, suggesting that stiffness can modulate YAP-mediated effects on cell speed and EMT. We indeed found that stiffer NRA substrata led to establishment of more aligned FAs (Supplementary Fig. 16a) and steeper spatial gradients of the cell speed values from the edge to the bulk regions of the cell sheets (Fig. 7b). The mathematical model predicted that decreasing substrate stiffness can lead to a gradual reduction of the area of YAP activation, shifting the activity closer to the sheet edges, thus decreasing the number of cells with fully active YAP (Fig. 7c). These results were indeed supported by the experimental analysis (Fig. 7d and Supplementary Fig. 16b), suggesting that both the topographic structure and effective stiffness of the underlying ECM can control YAP-dependent epithelial edge expansion and EMT (Supplementary Discussion and Supplementary Fig. 17). These results further supported the notion that mechanical cues can tightly control and modulate EMT events in expanding epithelial sheets.

The modeling analysis suggested a more refined view of the regulation of EMT by YAP-mediated topographically induced mechanical inputs (Fig. 7e). In wide marginal regions, enhanced cell migration and the overall sheet expansion can increase cell–ECM interaction (leading in particular to larger cell sizes, as seen, e.g., in Fig. 7e). This, in turn, can trigger the feedback interactions described above to enable a switch-like enhancement of YAP and WT1 activity and a strong decrease in E-cadherin expression, leading to loosening of cell–cell contacts and partial EMT in areas extending several cell diameters from the sheet edge. Further switch-like enhancement of cell migration speed can lead to elevated dynamic instability in the front of the sheet, leading to formation of particularly pronounced FLPs. This mechanism may provide a more mechanistic underpinning for the previous models of dynamic instability at the fronts of expanding epithelial sheets, resulting in FLP formation^60,61^. Due to a low engagement of E-cadherin, FLP-associated mechanical forces may not transfer into the submarginal regions, contrary to what is proposed for cells on flat surfaces^62,63^. E-cadherin decrease also can result in a reduced correlation length of collective cell migration and absence of swirling cell migration patterns that reflect the cohesiveness of the epithelial layer^64^. Rather, the apparently coordinated migration of the cells is supported by the continued contact guidance and mechanical input from the alighted matrix structures, which would promote, in a more cell-autonomous fashion, a switch-like increase in YAP activity and thus faster directed cell migration in extensive submarginal zones of the expanding sheets. The cells at the very edge of the sheet and particularly at the tips of FLPs, due to both lower E-cadherin expression and a large exposure to the cell-free boundary, would undergo a complete EMT and break free of the sheet. This model is consistent with the enhanced expression of the EMT markers, Twist and Slug, only in the marginal cells at the tips of FLPs and cell dissemination from this location (Supplementary Fig. 6b, c).

### Clinical relevance of mechanically induced EMT

**Fig. 8:**
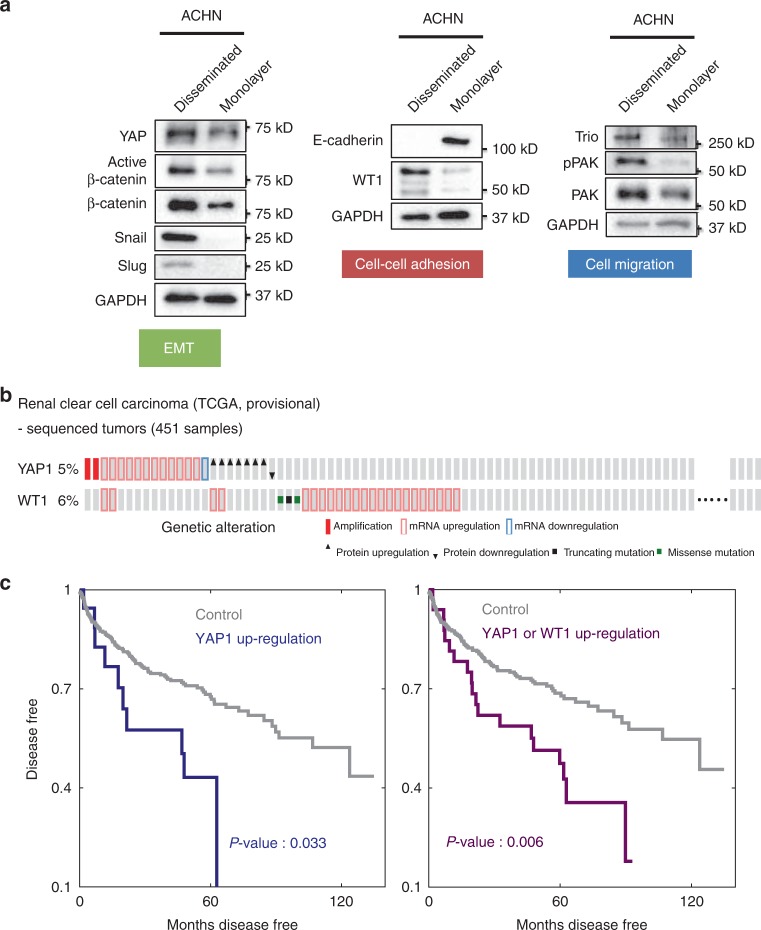
Potential role of the proposed mechanisms in the renal cancer. **a** Difference of signaling of renal cell carcinoma, ACHN, related to EMT, cell–cell adhesion, cell migration between disseminated cells, and cells remaining within the monolayer after dissemination elicited by mechanical cues. **b** Analysis of patient data, sequenced tumors (451 samples) of renal clear cell carcinoma, (TCGA, provisional) via cbioportal. Oncoprints show alterations in YAP1 and WT1 across a set of kidney renal clear cell carcinoma. *Z*-scores for mRNA expression (RNA Seq V2RSEM) and protein expression were 2.326 for 99% confidentiality (one tail). **c** Disease-free survival Kaplan–Meier estimate of upregulated YAP1 (left) and YAP1/WT1 (right) (see details in the “Methods” section)

Our results so far suggested that mechanical cues arising from the aligned fibrous structure of ECM can enhance collective cell spreading through a YAP-dependent EMT process even in normal renal epithelial cells. We hypothesized that this phenomenon might also influence aggressive spread in renal cancers. We thus explored the behavior of a renal cancer cell line, ACHN. When cultured on NRA, these cells displayed the behaviors similar to those of MDCK, including the EMT-like phenotype and cell dissemination, without any additional EMT triggers. We then contrasted the expression levels of various molecules implicated by our analysis in the NRA-driven EMT of MDCK cells in disseminated ACHN cells vs. the cells remaining in the monolayer. We found the results to be fully consistent with those displayed by the MDCK cells, for eight diverse proteins indicative of EMT and its feedback regulation by WT1 and TRIO (Fig. 8a). We then analyzed the patient data, querying the sequencing and expression profiles for 451 renal cancer samples contained in the TCGA database. We found that *YAP1* or *WT1* alterations were present in 5–6% of the cases (Fig. 8b). The mostly nonoverlapping pattern of the alterations of the genes coding for these proteins across the patient population suggested that an alteration of either of these two factors might be sufficient to impose a more aggressive phenotype, consistent with the regulatory networks proposed above. Therefore, we expected that altered expression of either one of them can be predictive of poor clinical outcome, with the joint *YAP1*-*WT1* signature increasing the predictive power of the analysis. We found that alterations in *YAP1* led to a much worse prognosis, with the predictive power gaining much higher significance, when both *YAP1* and *WT1* alterations were included in the signature (Fig. 8c). These results further support a critical role of YAP and the associated signaling proteins in aggressive spread of renal cancer or possibly other epithelial cancers, with possible implications for the disease progression.

## Discussion

We still know relatively little about the mechanisms of cellular responses to the mechanical topographic cues emerging from the matrix and tissue reorganization on multiple scales, from the curvature of blood vessels or muscle fibers^65–68^ to the subcellular-scale characteristic of ECM fibers^13,69^. The contact guidance provided by anisotropic mechanical cues associated with the alignment of the matrix fibers can help orient cell polarity and migration through mechanisms that are still only partially understood. By exerting forces on the matrix fibers, cells could potentially detect the anisotropic nature of the underlying substratum through experiencing a relatively higher resistance to compressive fiber deformation along the fiber direction vs. the relatively lower resistance to the bending and shifting the fibers in the orthogonal direction^70–72^. The anisotropic cues might also directly control the orientation of focal adhesion complexes and the associated cytoskeletal structures^73^. Whatever the mechanism, single- and collective cell behaviors can dramatically change, potentially influencing EMT and MET, accompanying a variety of normal and pathological processes. Here, we provide evidence for a dramatic, switch-like enhancement of partial and full EMT in normal and cancerous sheets of epithelial cells cultured on arrays of oriented fibers resembling in size and chemical input the natural ECM fibers. This outcome is similar in magnitude and biochemical nature to EMT triggered by more established chemical inputs, such as TGFβ, and acts in strong synergy with this, and potentially, other EMT-promoting chemical factors. This finding underscores the importance of diverse environmental inputs in the control of cell invasiveness, which can have profound effects even in the absence of genetic perturbations.

Our results suggest a mechanism of EMT regulation by anisotropic topography of the ECM-like cell adhesion substratum, which assigns a central role to feedback interactions involving a transcriptional co-regulator, YAP. We identified two feedback interactions through which YAP can control the EMT-like phenotype in response to nanotopographic stimulation: (a) suppression of E-cadherin expression through a WT1-dependent transcriptional regulation, leading to disruption of the epithelial integrity, and (b) enhancement of Rac1 activity and thus cell migration speed in a Merlin- and small GTPase-dependent fashion. These feedback interactions may be triggered by other YAP-modulating inputs, including those directly controlling cell geometry, although cell elongation alone might lead to inactivation rather than activation of YAP, as suggested by micro-contact printing of ECM cues^74^, but contrary to the effects observed here. These findings and the findings in Sagar et al.^47^ suggest that YAP activation can be a central regulator of both single- and collective cell migration in the presence of complex mechanical cues supplied by ECM, providing further insight into the general mechanisms of contact guidance. The combined effects of the two feedback loops create a robust switch that can be modulated by other inputs, including additional mechanical cues (e.g., ECM stiffness or physical confinement) implicated in invasive cancer spread. The biochemical switches modulated by YAP suggest a mechanism underlying regulation of the dynamical transition between collective and individual cell migration analogous to a phase transition in physical and chemical systems, e.g., during binary-mixture solidification^75^. In particular, our results suggest that decreasing ECM rigidity might partially negate the EMT-inducing effect of the aligned ECM fiber structure, providing a potential for new interventions into the aggressive spread of normal and cancerous cells.

The functional WT1-YAP interaction identified here has interesting implications for understanding of developmental and pathogenic processes. First, since WT1 has also been studied as a crucial regulator of mesenchymal–epithelial transition (MET) (a process commonly seen as inverse to EMT), our results suggest that both EMT and MET can be controlled by the same molecular network in a switch-like fashion, shedding further light on MET events during Wilms tumor progression and normal kidney development. In particular, they suggest a mechanistic explanation for the prior results, implicating YAP in kidney development and for the consequences of YAP dysregulation in anaplastic Wilms tumors^76,77^. Furthermore, our results elucidate the interplay between YAP and WT1 in the EMT-like events during epicardial development^78^. We also show that in contrast to the regulation of Merlin during collective cell migration on flat substrata^37^, mutual regulation between YAP and Merlin becomes a critical part of enhanced and more individualized cell migration in the presence of additional mechanical inputs. The feedback nature of this interaction can help reconcile our results here and in Sagar et al.^47^, with the prior observations placing TRIO upstream of YAP^79^. In particular, our results suggest that TRIO can be thought of being both upstream and downstream of YAP, by being a part of a feedback linking YAP to Rac1. Overall, these mechanistic insights provide a framework for understanding the two components of a successful EMT process: increased cell migration and decreased cell linkage, through two feedback mechanisms, both modulated by YAP and triggered by mechanical stimuli.

The feedback nature of the mechanical control of EMT elucidated in this study can be critical both in normal processes involving EMT and in cancer progression. Our data suggest that the findings made in normal renal epithelial cells translate to renal cancer, permitting identification of a novel clinical signature, relying on the analysis of alterations in both YAP and WT1, which together account for a significantly poorer prognosis for patients with renal cancers. These results argue that YAP-WT1 interaction can promote renal cancer progression. We therefore propose that the methods of analysis described in this study can be used to further investigate EMT and invasive cell spread in renal cancers, and assist in furthering precision medicine approaches in the clinic.

Mechanical stimuli can stem from a variety of sources, encompassing the topography, rigidity, and other features of ECM composition and organization. Our results suggest how YAP can integrate these diverse mechanical cues and translate them into regulation of EMT. This could occur in normal and cancerous epithelial sheets following matrix reorganization, with a dramatic switch-like change in the signaling profiles, leading to enhanced cell speed and loosening cell–cell contacts, the processes that can be conserved in diverse physiological and pathological settings.

## Methods

### Fabrication of the NRA substrata

The nanostructured substrata (width: 800 nm, spacing: 800 nm, and height: 800 nm) were fabricated by capillary-force lithography with UV-curable polyurethane acrylate (PUA, Minuta Technology, South Korea) polymer having the rigidity value of 0.1 GPa, unless indicated otherwise. To fabricate a flexible polymer mold, a precursor of UV-curable polymer was drop-dispensed onto the prefabricated silicon master with large areas of 25 × 25 mm^2^. Then a polyethylene terephthalate (PET) (SKC Inc., South Korea) film with thickness of 50 μm was brought into conformal contact onto the polymer resin. After removing air bubbles with a roller, UV (*λ* = 250–400 nm) was irradiated for few tens of seconds for the cross-linking. The flexible and transparent mold with PET support was obtained after peeling off from the master, leaving behind regularly spaced nanogrooves, i.e., NRA. For the polymer nanogroove patterns on the slide glass-sized coverslips (50 × 24 mm, Thermo Fisher), the same replication process was performed onto a cleaned coverslip, using the replicated PUA pattern as a mold. The flat surfaces were generated as a control experimental set with the same procedure except no master. To study the effect of rigidity, we also used different prepolymers for fabrication as follows: PU elastomer (MINS 311 RM) for 10 MPa was purchased from Minuta Technology (Korea). Hard PU (NOA83 H) for 1 GPa was purchased from Norland Optical Adhesive Inc. (NY, USA). The detailed information of soft and intermediate PU materials can be found elsewhere^80^. These patterns were assembled onto four multiwell chambers (Nunc® Lab-Tek® Chamber Slide^TM^ system) after removing premounted slide glasses on their bottom. We chose the dimensions of NRA based on the dimension that led to the most aggressive expansion shown on variable-density ridged arrays. An example of an experiment examining differential speed regulation on such surfaces is shown in Supplementary Fig. 1.

### Analysis of epithelial cell sheet migration

We purchased MDCK cells and GFP-α-tubulin and GFP-F-actin-transfected MDCK cells from Marinpharm GmbH and ACHN from ATCC (CRL-1611). Cells were cultured in Dulbecco’s modified Eagle’s medium (Gibco) supplemented with 10% fetal bovine serum (Gibco), 50 U ml^−1^ penicillin, and 50 μg ml^−1^ streptomycin (Invitrogen) at 37 °C, 5% CO_2_, and 90% humidity. These were split 1:4 after trypsinization for passaging every 2–3 days. Glass coverslips covered with the NRA nanotopographic substrata were preglued onto the bottom surface of the custom-made MatTek dish (P35G-20-C) or four multiwell chambers (Nunc® Lab-Tek® Chamber Slide^TM^ system). Then, cells were replated on the patterns precoated with 30 mg/ml of collagen type I (Invitrogen, A1048301) for 3 h or 20 μg/ml of fibronectin (Sigma, F0896) and incubated for up to 12 h for the generation of monolayer sheets. More specifically, on collagen type I or fibronectin precoated substrata, a PDMS insert (Supplementary Fig. 1a) that has two 7- × 2-mm rectangular holes was deposited, and 2.0 × 10^5^ cells in 50 μL of medium were seeded into each hole. After incubating for about 12 h, the insert was gently removed with tweezers. For the perturbation of the signaling pathway related to cell migration, 10 mM of Y27632 (Sigma-Aldrich), 100 mM of NSC23766 (Tocris), 5 μg/mL of Mitomycin C (Sigma-Aldrich), 10 μM of ITX3 (Tocris), 10 μg/mL of anti-E-cadherin antibody (Sigma-Aldrich, U3254), and 5 ng/mL of recombinant TGFβ1 (R&D Systems) were added. To show cell-density dependence of nuclear localization of YAP and WT1, 5 × 10^5^ cells/cm^2^ of cells were seeded and the cell density was twofold serially diluted three times. For immunofluorescence staining, the following samples were chosen: dense (5 × 10^5^ cells/cm^2^) and sparse (1.25 × 10^5^ cells/cm^2^), and for immunoblotting, we used all four different cell-density samples.

### Lentiviral transfection

To generate YAP-knockdown MDCK cells, MDCK cells were infected with lentivirus containing shRNA-targeting YAP (pLKO1-shYAP1, Addgene, 27368) or Human Mission TRC1 sequence-verified YAP shRNA lentiviral plasmid vectors (TRCN0000107265, Sigma-Aldrich) and control plasmids, pLKO1 empty vector (Sigma-Aldrich, SHC001V). First, HEK293T cells were transfected with plasmids, vesicular stomatitis virus glycoprotein (VSVG) (Addgene, 8454), and D8.2 dvpr (Addgene, 8455) with FuGENE 6 (Roche, 11814443001) and the medium was changed after 12 h. The supernatant collected at 72 and 96 h after transfection was transferred to MDCK culture with polybrene (Sigma-Aldrich) at a final concentration of 5 μg/mL. Cells were selected in culture medium containing 2 μg/mL of puromycin (Sigma-Aldrich).

### Small-interfering (siRNA) RNA transfection

siRNA transfection was performed with siRNA reagent system (Santa Cruz, sc-45064) following the manufacturer’s instructions. Briefly, cells were replated in six-well plates to 40% confluence in antibiotic-free 10% FBS-containing medium. For each transfection, 6 μl of WT1 siRNA (Santa Cruz, sc-36846), TAZ siRNA (Thermo Fisher, 122501), and unconjugated control siRNA-A (Santa Cruz, sc-37007) in 100 μl of transfection medium (Santa Cruz, sc-36868) were mixed with 6 μl of transfection reagent (sc-29528) in another 100 μl of transfection medium. The mixtures were incubated for 30 min at room temperature. Each well was washed with 2 ml of transfection medium once and then filled with a prepared reagent mixture plus 0.8 ml of transfection medium. After incubation overnight, an additional 1 ml of medium supplemented with 20% FBS with 2% antibiotics was added to each well for another 24 h. The transfection mixture was removed and replaced with normal growth medium on the following day. All experimental measurements were performed 24 h following replacement of the medium. The sequence information of siRNAs will be available upon request.

### Plasmid and transfection

The plasmid of pcDNA-Flag-YAP1 was purchased through Addgene (18881) and transfected into cells using Lipofectamine 3000 (Thermo Fischer) according to the manufacturer’s instructions. The protein was assessed 24 h later by western blotting.

### Immunofluorescence staining

The samples on the nanofabricated coverslip were fixed with ice-cold 4% paraformaldehyde for 20 min, washed two times with phosphate-buffered saline (PBS), and permeablized with 0.1% Triton X-100 in PBS for 5 min. After washing with PBS, cultures were blocked with 10% goat serum for 1 h, and then incubated with primary antibodies against Vinculin (1:200, Sigma-Aldrich), E-cadherin (1:200, Cell Signaling, 3195), phospho-Myosin Light Chain (1:200, Cell Signaling, 3674), γ-tubulin (1:100, Abcam, ab11321), YAP (1:100, Cell Signaling, 4912 and Santa Cruz, sc-15407), active β-catenin (1:100, Millipore, 05-665), Vimentin (1:100, Abcam), Slug (1:100, Cell Signaling, 9585), Twist (1:100, Santa Cruz, sc-15393), Vimentin (1:100, Abcam, ab24525), and WT1 (1:100, Santa Cruz, sc-192) for 3 h at room temperature. After washing with secondary antibodies and Alexa Fluor 594 conjugated phalloidin (1:40, Molecular Probes) and Hoescht (Invitrogen), cultures were incubated for 1 h at room temperature. The slides were mounted with an anti-fade reagent (SlowFade gold, Invitrogen) and taken by an inverted microscope (Zeiss Axiovert 200 M) with an X40 oil immersion objective (Zeiss, 1.6 NA).

### Immunoblot analysis

To ensure availability of sufficient amounts of protein samples for immunoblotting in each experiment, we used 30 samples of initially confined epithelial monolayers of MDCK cells through attaching the custom-made PDMS stencils with multirectangular holes as confining surfaces on the same coverslips (25 × 25 mm^2^, Thermo Fisher, 12546), with or without nanostructured surfaces (see Supplementary Fig. 1 for details). This high number of monolayers used per experiment allowed us to collect sufficient numbers of cells for biochemical analyses. We collected and homogenized cells in RIPA buffer (Thermo Fisher Scientific) with protease inhibitor cocktail (Thermo Fisher Scientific), and then centrifuged them at 12,000 × *g* at 4 °C for 20 min, followed by collection of the supernatant. Nuclear protein extraction was performed with Thermo Scientific Pierce NE-PER nuclear and cytoplasmic extraction reagents following the manufacturer’s protocol (Thermo Fisher Scientific, 78833). For Rac1 pull-down assay, we used Rac1 pull-down biochemical assay kit (Cytoskeleton Inc., BK035) and followed the manufacturer’s protocol. Protein concentration was quantified using the BCA assay kit (Thermo Fisher Scientific, 23227). Protein samples were subsequently diluted with sample buffer, heated at 70 °C for 10 min. These samples were separated on a 4–20% w/v SDS PAGE gel (Bio-Rad), and transferred to a nitrocellulose membrane (Bio-Rad). This membrane was blocked for 1 h in blocking solution, TBST (10 mM Tris, pH 8.0 and 0.1% v/v Tween 20), supplemented with 5% BSA (Bio-Rad) and incubated in 1:500 diluted solution of Snail-specific antibody (Cell Signaling, 3879), 1:500 diluted solution of anti-E-cadherin antibody (Cell Signaling, 3195), 1:1000 diluted solution of anti-Myosin Light Chain antibody (Abcam, ab48003), 1:500 diluted solution of anti-phospho-Myosin Light Chain antibody (Cell Signaling, 3674), 1:1000 diluted solution of anti-YAP antibody (Cell Signaling, 4912 and Santa Cruz, sc-15407), 1:1000 diluted solution of anti-phospho-YAP antibody (Cell Signaling, 4911), 1:1000 diluted solution of anti-active β-catenin antibody (Millipore, 05-665), 1:1000 diluted solution of anti-β-catenin antibody (Santa Cruz, sc-7963), 1:100 diluted solution of anti-TRIO antibody (Santa Cruz, sc-28564), 1:1000 diluted solution of anti-phospho-PAK antibody (Cell Signaling, 2601), 1:1000 diluted solution of anti-PAK antibody (Cell Signaling, 2602), 1:1000 diluted solution of anti-phospho-Merlin antibody (Cell Signaling, 9163), 1:1000 diluted solution of anti-Merlin antibody (Cell Signaling, 6995), 1:1000 diluted solution of anti-Angiomotin antibody (Santa Cruz, sc-98803), 1:1000 diluted solution of anti-WT1 antibody (Santa Cruz, sc-192), 1:2000 diluted solution of anti-laminB1 antibody (Abcam, ab28129), and 1:1000 diluted solution of anti-GAPDH antibody (Abcam, ab9483) in blocking solution at 4 °C overnight. After washing (three times for 10 min) in TBST, goat anti-rabbit and anti-mouse secondary antibodies for Odyssey® western blotting (Li-Cor) or HRP-conjucated secondary antibodies (Pierce, 31160 (anti-mouse) and 31188 (anti-rabbit)) were treated. Finally, after washing with TBST, Odyssey® CLx infrared imaging system (Li-Cor) or Bio-Rad gel-imaging system, ChemiDoc^TM^ XRS + (Bio-Rad) with Pierce ECL Western Blotting Substrate (32106) was applied to blotting.

### Co-immunoprecipitation analysis

Co-immunoprecipitation (co-IP) was performed using the Thermo Scientific Pierce co-IP kit following the manufacturer’s protocol (Thermo Fisher Scientific, 26140). Briefly, 60 μl of anti-YAP antibody (Santa Cruz, sc-15407), 60 μl of anti-Angiomotin antibody (Santa Cruz, sc-98803), and 30 μl of IgG antibody (Santa Cruz, sc-2027) were first immobilized for 2 h using AminoLink Plus coupling resin. The resin was then washed and incubated with 200 μl (500 μg of proteins) of lysate harvested using IP/lysis buffer in the kit and precleaned with control agarose resine for 1 h. After incubation, the coupling resin was again washed and protein was eluted using elution buffer. Samples were analyzed by immunoblotting.

Chromatin immunoprecipitation analysis. Chromatin immunoprecipitation (ChIP) was performed using the Thermo Scientific Pierce agarose ChIP kit following the manufacturer’s protocol (Thermo Fisher Scientific, 26156) or SimpleChIP® Enzymatic Chromatin IP Kit (Cell Signaling, 9003). In total, 1 × 10^6^ cells per well were cross-linked with formaldehyde and their lysates were digested with 1 U of MNase for 5 min in a 37 °C water bath. Then, we immunoprecipitated digested lysates with 5 μL of anti-YAP antibody (Santa Cruz, sc-15407X), anti-WT1 antibody (Santa Cruz, sc-192X), anti-RNA polymerase II antibody, and 1 μL of rabbit IgG. All DNA in digested lysates have an average length of 0.25–1 kb. PCR amplification was performed in 50 μL of mixture with Q5 high-fidelity DNA polymerase (NEB, M0491) and specific primers. The ∼150-bp fragment of canine E-cadherin promoter was amplified with the primers 5′-CCCGCCGCAGGTGCAGCCGCAGC-3′ (direct) and 5′-GAGGCGGCGCGAGGCCGGCAG-3′ (reverse)^41^. Also, we used SimpleChIP® Human CTGF promoter primers (Cell Signaling, 14927) and GAPDH promoter primer in the kit (Thermo Fisher Scientific, 26516) as control primers. PCR was carried out in the following program: 25 cycles at 94 °C for 40 s, 65–68 °C for 40 s, and 72 °C for 40 s. The amplified DNA was separated on 1% agarose gel and visualized with ethidium bromide.

### Luciferase assay

For the generations of the corresponding cells (control, YAP^KD^, and WT1^KD^ MDCK cells), we followed the protocol in lentiviral transfection and small-interfering (siRNA) RNA transfection. Cells were cultured in 96-well plates and were co-transfected with reporter vectors. We used Lipofectamine 3000 (Thermo Fisher) according to the manufacturer’s instructions for the transfection. The *CDH1* (E-cadherin) promoter luciferase activity reporter vectors were purchased from Addgene (#42081). Also, as a control reporter, we used pRL-*Renilla* luciferase control reporter vectors (E2231, Promega). For the assay, we used luciferase assay kit (E1500 and E2810) following the manufacturer’s protocols.

### RNA extraction and quantitative real-time PCR

Total RNA was extracted using mirVana^TM^ miRNA Isolation Kit (Applied Biosystems, AM1560) according to the manufacturer’s instructions. One microgram of purified RNA was reverse transcribed using the iScript™ Reverse Transcription Supermix (Bio-Rad). Quantitative real-time PCR using TaqMan^TM^ Fast Advanced Master Mix (4444556, Thermo Fisher Scientific) was performed with CFX384 Touch^TM^ Real-Time PCR Detection system and the included data analysis software. YAP1 primer (Cf02644450_m1, Thermo Fisher), Snai1 primer (Cf02705362_s1, Thermo Fisher Scientific), VIM primer (Cf02668853_g1, Thermo Scientific), CDH1 primer (Cf02697525_m1, Thermo Scientific), and GAPDH primer as a loading control (Cf04419463_gH, Thermo Scientific) were used for analysis.

### Time-lapse microscopy

Images were acquired after removing stencils, i.e., after allowing free expansion of cell sheets. The custom-made multiwell chamber integrated with the topographically patterned substratum was mounted onto the stage of a motorized inverted microscope (Zeiss Axiovert 200 M). The microscope was equipped with a Cascade 512B II CCD camera that has the environmental chamber containing. Phase-contrast images of cells were automatically recorded using the Slidebook 4.1 (Intelligent Imaging Innovations, Denver, CO) for 6 h at 5-min intervals.

### Cell tracking and quantification

We tracked cells manually in every image taken every 20 min in a time-lapse imaging for 6 h, with the aid of a customized MATLAB (The MathWorks, Natick, MA) code. The positions of cells with respect to time after identifying and tracking individual cells were used to analyze the migratory behavior. Cell migration speed was calculated by averaging displacements per each time interval, 20 min. The DT ratio was defined as the ratio of the shortest distance between the initial location of an individual cell and its final location to the length of the whole trajectory of the cell. We considered cells in the range of 70 μm from the sheet edge as marginal cells. The velocity correlation length refers to the distance where the correlation function reaches zero. We calculated the velocity correlation function in the parallel direction to epithelial expansion (flat surfaces) and the direction of a grooved structure (NRA), following Vedula et al.^81^ The function was calculated from the trajectories of cells in the range from 70  to 300 μm from the sheet edge of the monolayer (the range chosen based on YAP translocation patterns). Statistical analyses were performed using unpaired two-sided Student’s *t*-tests. Every experimental condition was analyzed in duplicate and at least 80 cells were tracked in each data replicate.

### Analysis of clinical samples

Cbioportal was utilized to analyze the clinical data of renal clear cancer patient samples for evaluating YAP and WT1 as prognostic indicators. We investigated the database of sequenced tumors (451 samples) of renal clear cell carcinoma (TCGA, provisional). *YAP1* and *WT1* were queried in the database to explore the genetic alterations and their related genomic profiles (mutations, copy-number variation, mRNA expression, and protein expression) of each sample in the database. mRNA (RNA Seq V2RSEM) and protein expression levels were subject to threshold-based selection to sort out the samples with statistically significant alterations. Specifically, *Z*-scores were >2.326 for 99% confidentiality (one tail). To evaluate the effects of YAP and WT1 on cancer progression, we separated the samples into a group with genetic alterations hypothesized to increase YAP or WT1 activity (amplification in the copy number, increased RNA, and protein expressions) and the other group without such alterations. The clinical information for grouped patient samples was obtained from the TCGA database via Cbioportal (see above). We provide the grouped data as a Supplementary data file. We then used the Kaplan–Meier analysis and log-ranked P test to estimate the prognosis values of YAP, comparing the disease-free periods of patients who had *YAP1* alterations and those of patients who had *YAP1* or *WT1* alterations from those of patients who did not have them.

## Supplementary information

Supplementary information

Description of Additional Supplementary Files

Supplementary movie 1

Supplementary movie 2

Supplementary movie 3

Supplementary movie 4

Supplementary movie 5

Supplementary movie 6

Supplementary movie 7

Supplementary movie 8

Supplementary movie 9

Supplementary movie 10

Supplementary movie 11

Source Data

## Data Availability

The data that support the findings of this study are available from the corresponding author upon request. The source data underlying Fig. 8c are provided as a Supplementary Data file. All raw images of immunoblotting are provided as Supplementary Fig 18.
