# Supplementary material for: Switch-like enhancement of epithelial-mesenchymal transition by YAP through feedback regulation of WT1 and Rho-family GTPases
Source: Nat Commun. 2019 Jun 26;10:2797. doi: 10.1038/s41467-019-10729-5 (PMC6594963; doi:10.1038/s41467-019-10729-5)
Supplement: Supplementary file 1 — Supplementary information [file 41467_2019_10729_MOESM1_ESM.pdf]

SUPPLEMENTARY INFORMATION

Levchenko *et al.*

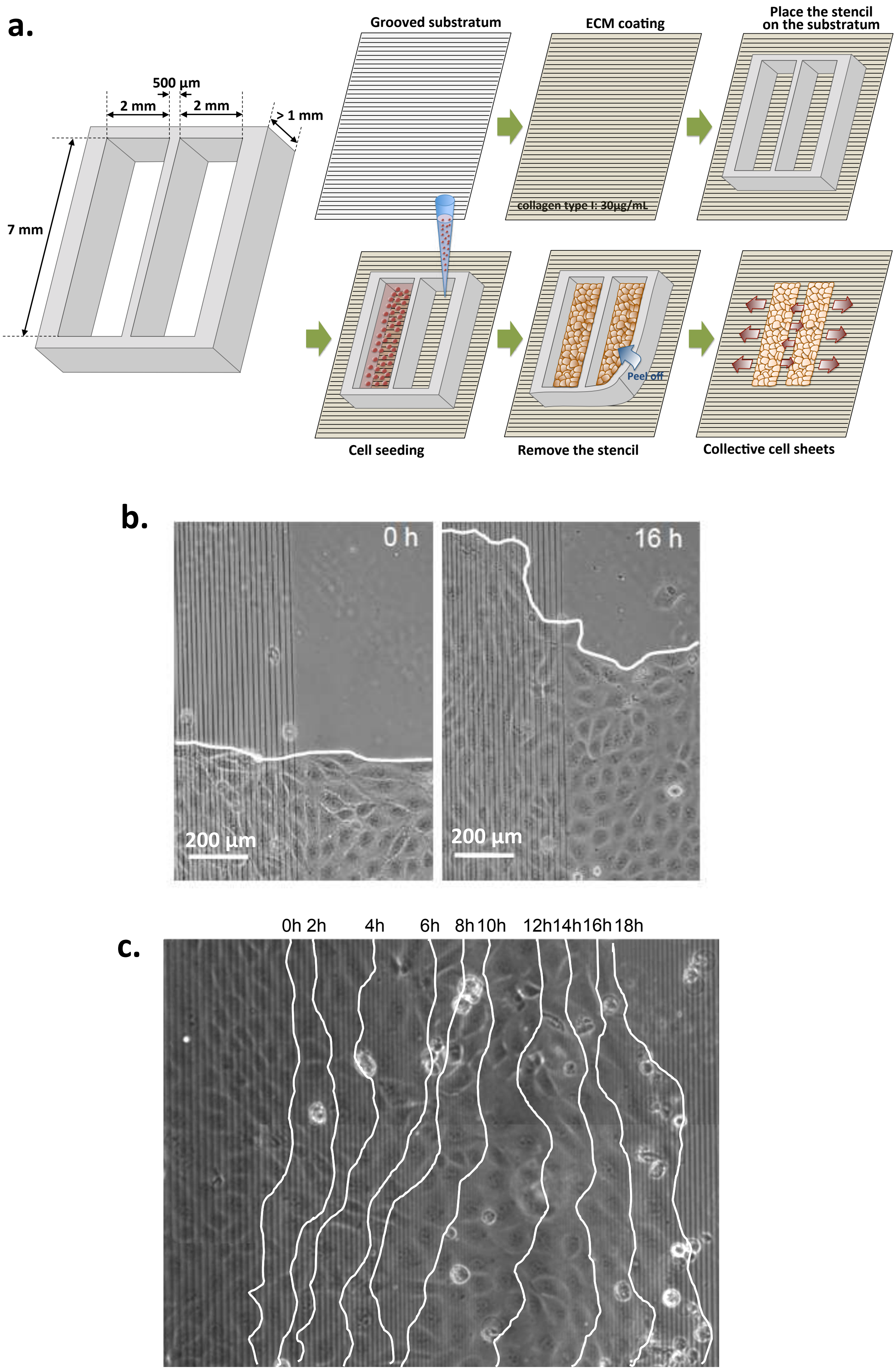
a.
Grooved substratum
ECM coating
Place the stencil
on the substratum
500 µm
2 mm
2 mm
> 1 mm
7 mm
collagen type I: 30µg/mL
Peel off
Cell seeding
Remove the stencil
Collective cell sheets
b.
0 h
16 h
200 µm
200 µm
c.
0h 2h 4h 6h 8h 10h 12h 14h 16h 18h

Supplementary Figure 1. Procedure for generation of epithelia sheets on NRA. **a.** Microstencil of PDMS were prefabricated and used to corral the cells and define the initial shape of the sheet. NRA surfaces were coated with 30 μg/mL of collagen type I for 3 hours. After aspirating the solution of collagen type I, microstencils defining the size of the initial epithelial sheets were bonded to the nano-topographically defined substratum. In each rectangular hole of a microstencil, 2.0×10^5 cells diluted in 50 μL of media were seeded. After full cell attachment to the substratum overnight, the microstencils were peeled off. Thereafter, the expansion of epithelial cell sheets was tracked using microscopy. **b.** Collective cell migration for 16 hours on flat and NRA substrata in a single experiment. **c.** Epithelial sheet expansion perpendicular to the direction of the nano-ridge array of NRA for 18 hours, showing a lack of FLPs and a lower degree of expansion vs. that in the direction of the ridges.

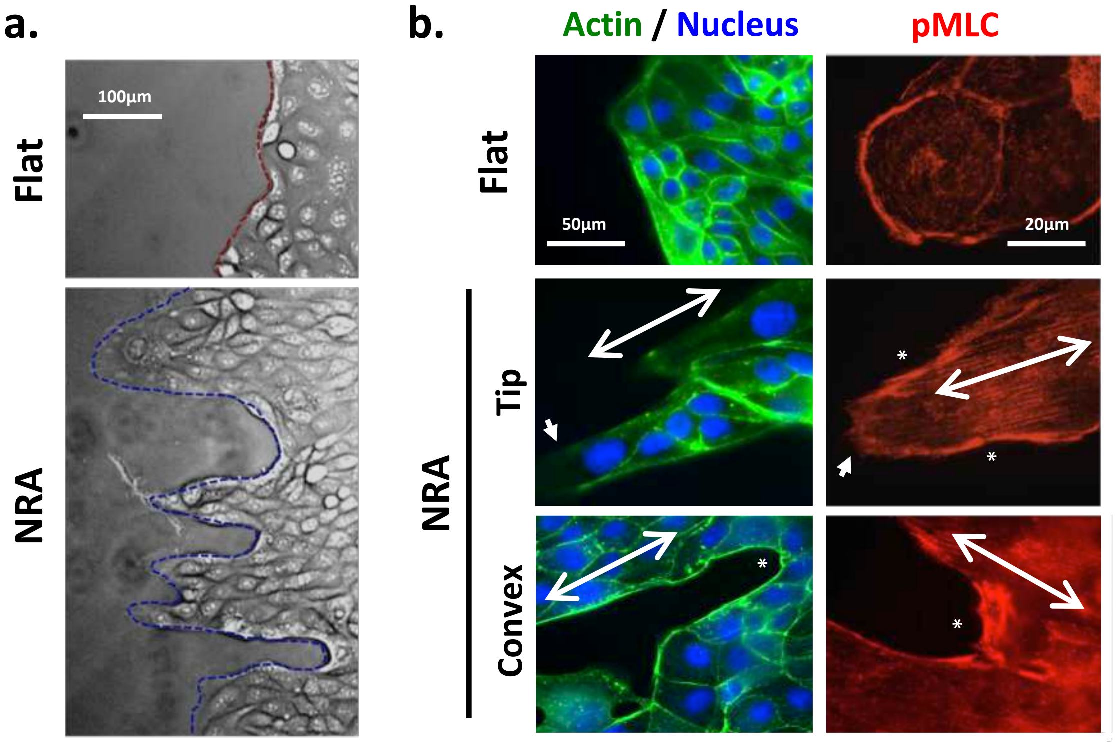

Supplementary Figure 2. Analysis of cytoskeleton within FLP forming on NRA. a. An example of FLPs formed on an NRA. **b.** Immunofluorescence staining for actin of cells on a flat and anisotropic surfaces (left). An arrow marks lack of cortical actin at the tip of FLPs and an asterisk points out concentrated actin at the convex region of the edge. Phosphorylated myosin light chain (pMLC) at the frontal periphery of FLPs on a flat surface and NRA (right). An arrow indicates weak pMLC at the tip of a FLP on NRA and asterisks point at concentrated pMLC at the sides of the FLP. Double-sided arrows indicate the direction of groove/ridge arrays.

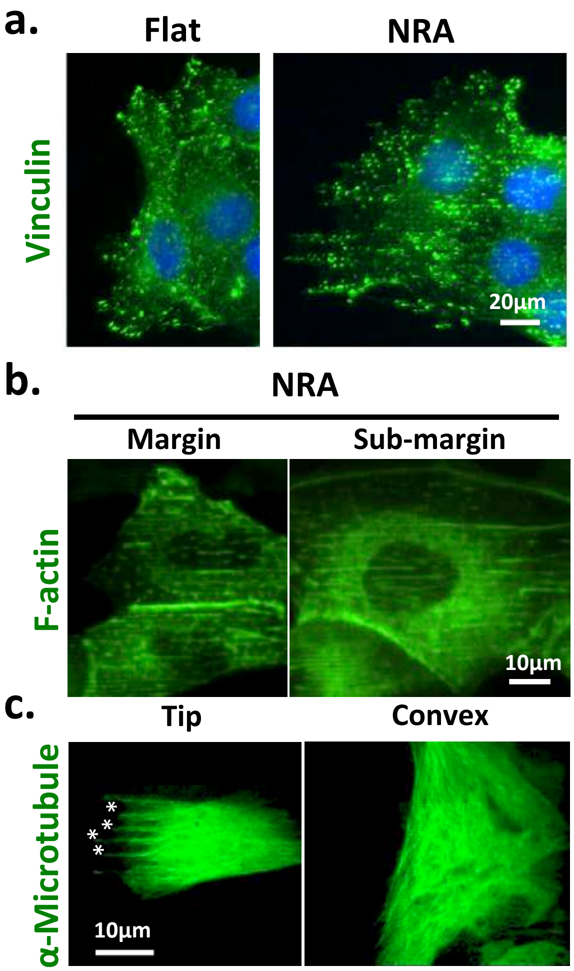

Supplementary Figure 3. Focal adhesions and cytoskeleton in epithelial cell layers on NRA. a. Images of cells immuno-stained for vinculin on a flat surface and NRA **b.** A snapshot from a live-cell imaging experiment analyzing GFP-F-actin transfected cells in the marginal and sub-marginal region of an epithelial sheet. **c.** A snapshot from a live-cell imaging experiment of GFP-α-tubulin transfected cells at the tip and the convex regions in the expanding boundary of an epithelial cell sheet. Asterisks indicate aligned microtubule structures at the tip of FLPs along the direction of topographical cues.

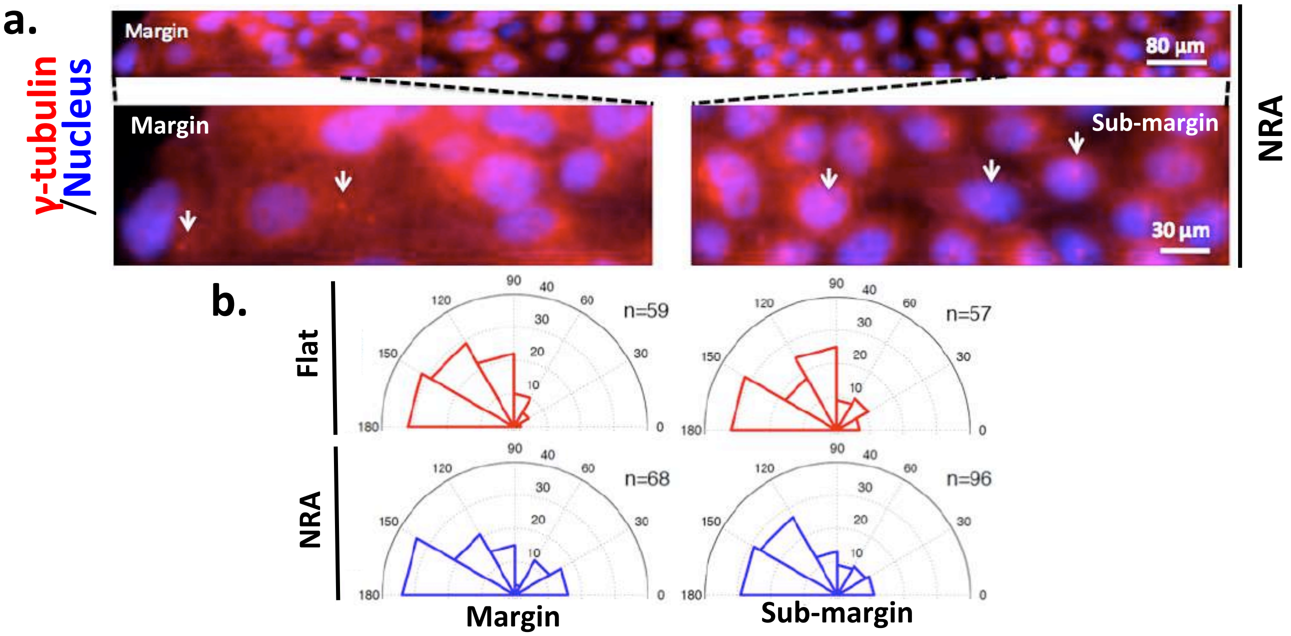

Supplementary Figure 4. Polarization of cells in the epithelial monolayers on flat surfaces and NRA. a. Immunofluorescence staining of Microtubule-organizing center (MTOC), a polarization marker, in an epithelial cell layer cultured on NRA. **b.** The histogram of angles between the axes connecting the nuclei to MTOCs and the direction of ridges on NRA in cells located in marginal or sub-marginal regions of epithelial cell sheets cultured on flat substrata and NRA.

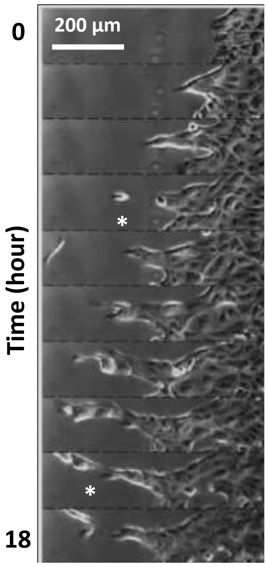

Supplementary Figure 5. Example of disseminated cells emerging from the advancing cell sheet cultured on NRA for 18 hours. Asterisks indicate the disseminated cells

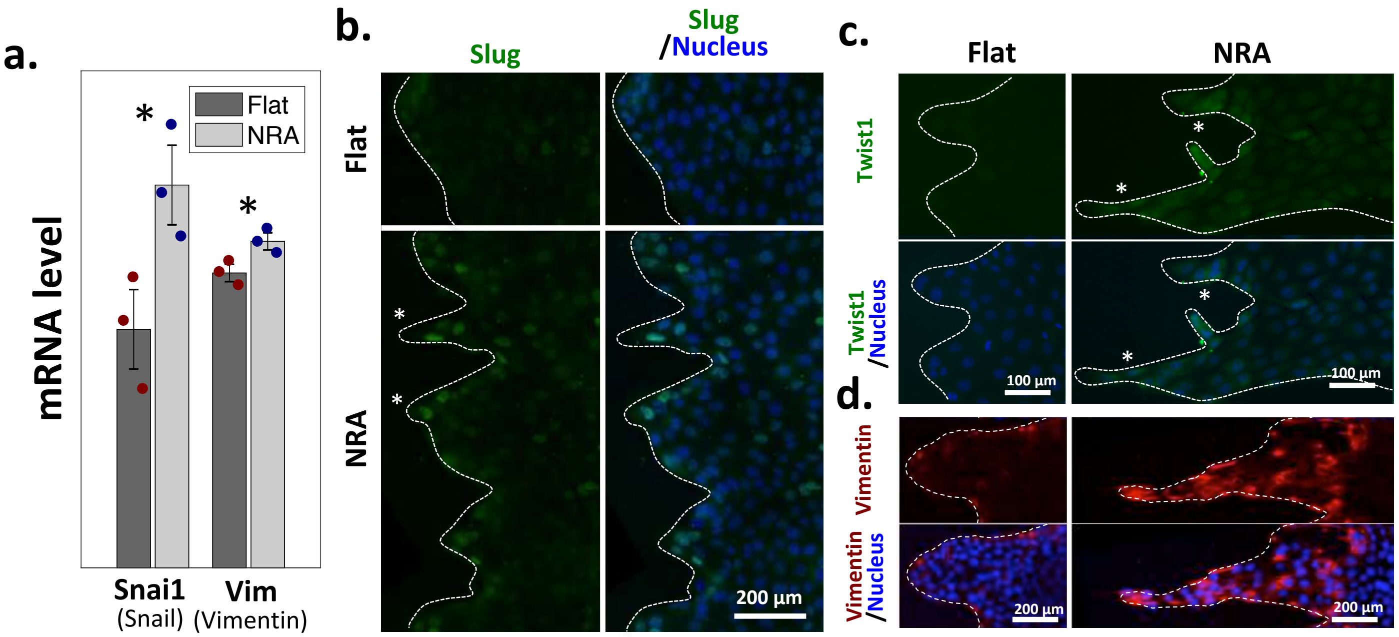

Supplementary Figure 6. Molecular characterization of mechanically induced EMT. a. mRNA expression of *Snai1* and *VIM* of cells cultured on flat surfaces vs. NRA. All error bars are S.E.M (n = 3, * = statistical significance of mRNA expression between cells on flat vs. NRA. *$P<5x10^{-2}$, all two-sided Student's t-test) **b.** Immunofluorescence staining for Slug and **c.** Twist expression in FLPs in cells cultured on flat and NRA surfaces. Asterisks mark exclusive expression of EMT markers on FLPs on NRA. Dashed lines indicate the sheet edge. **d.** Vimentin staining proximal to the edge of a cell sheet on a flat substratum and NRA. All samples were fixed after 8 hours to remove stencils.

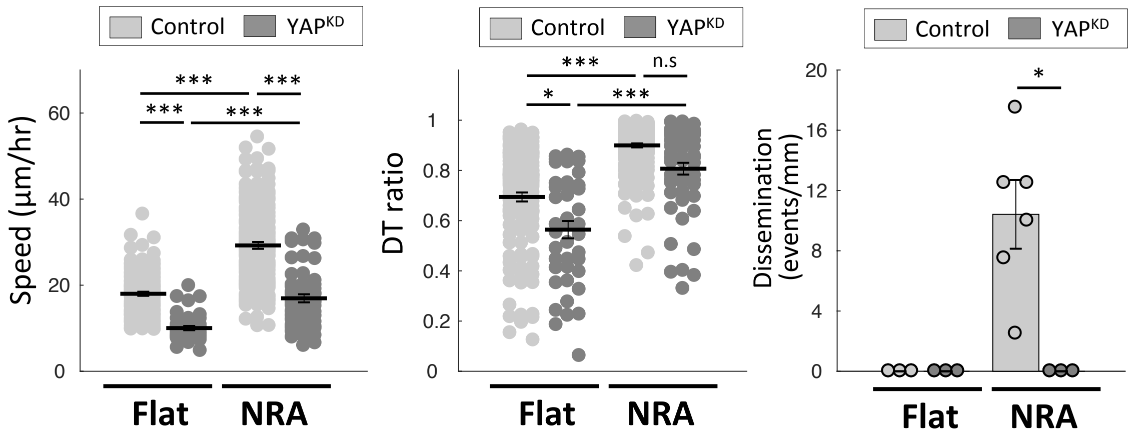

Supplementary Figure 7. Cell migration speeds (left), DT ratio (middle) and the number of disseminated cells (right) of control and YAP^{KD} cells in the marginal regions on flat vs. NRA surfaces. All error bars are S.E.M. (n.s = no significance, *$P<5x10^{-2}$, **$P<1x10^{-2}$, and ***$P<5x10^{-3}$, all two-sided Student's t-test).

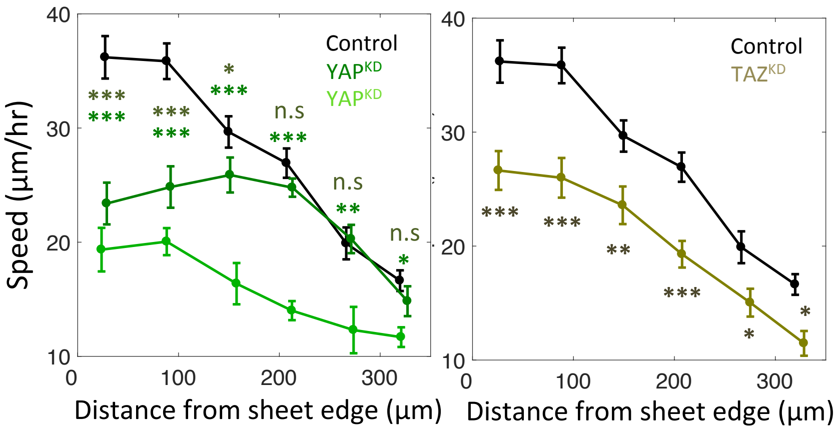

Supplementary Figure 8. Cell migration speed of in YAP^{KD} (left) and TAZ^{KD} epithelial cell sheets (right) as a function of the distance from the sheet edge on fibronectin-coated NRA. All error bars are S.E.M. (* = Statistical significance of control vs. YAP^{KD} or TAZ^{KD} cells, n.s = no significance, *P<0.05, **P<5x10^{-4}, and ***P<5x10^{-6}, all two-sided Student's t-test).

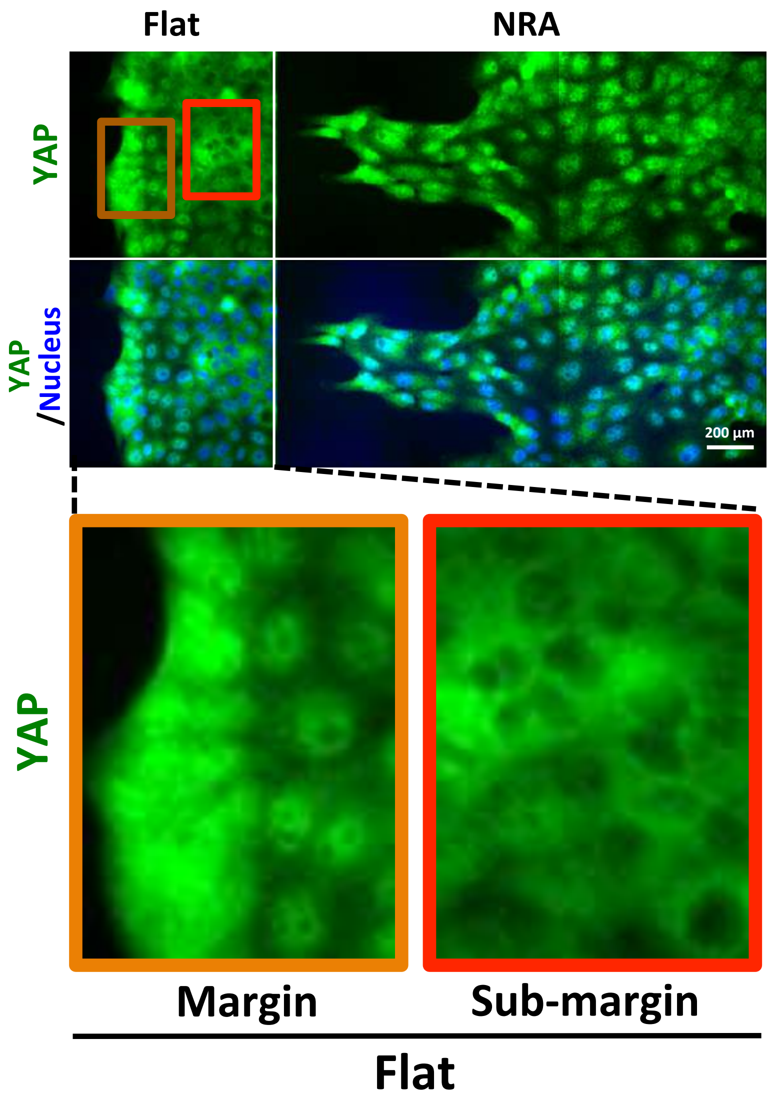

Supplementary Figure 9. Immunofluorescence staining of YAP of cells on fibronectin coated flat and NRA substrata. In the marginal zone and FLPs of sheets on flat surfaces pre-coated with fibronectin, YAP was primarily localized in nuclei (brown boxes), whereas it was localized in the cytosol of sub-marginal cells (red boxes). On NRA pre-coated with fibronectin, regardless of location of cells within the epithelial layer, either marginal or sub-marginal regions, YAP was localized in nuclei.

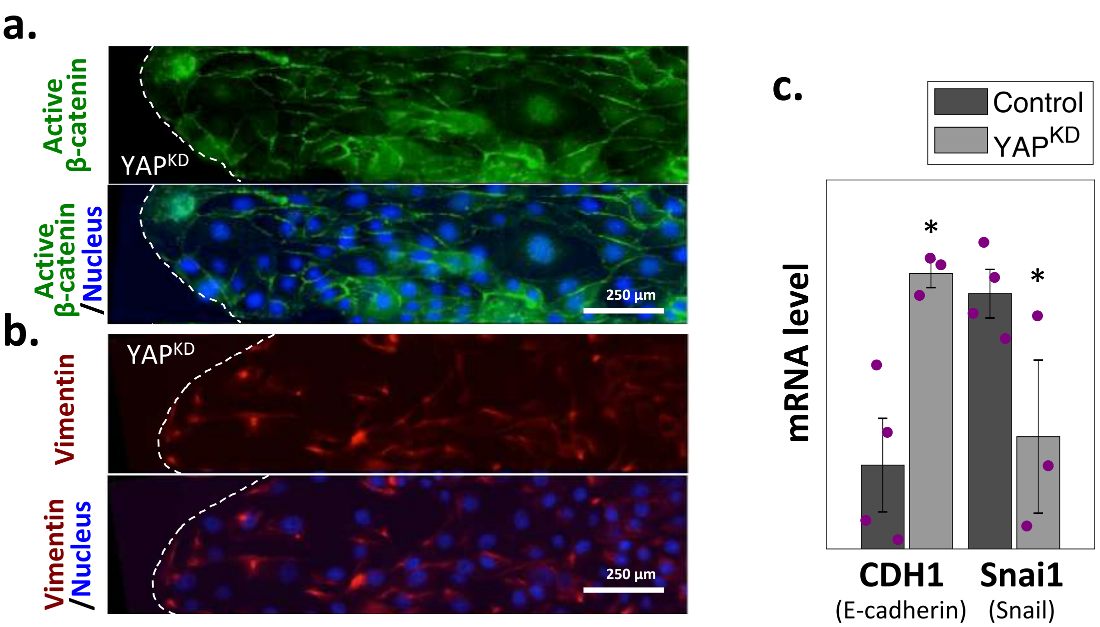

Supplementary Figure 10. Suppressed EMT characteristics in YAP^{KD} cells on NRA. a. Decreased localization of β-catenin in the nuclei and **b.** expression of vimentin (bottom) in the marginal regions of YAP^{KD} cell sheets cultured on NRA. **c.** mRNA expression of *CDH1* and *Snai1* of control vs. YAP^{KD} cells cultured on NRA. All error bars are S.E.M (n = 3). (* = statistical significance of mRNA expression between cells on flat vs. NRA. $*P<5x10^{-2}$, all two-sided Student's t-test).

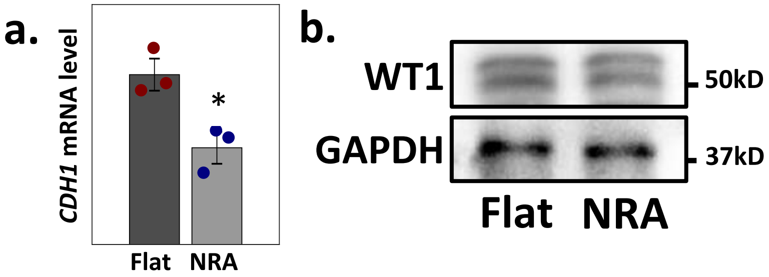

Supplementary Figure 11. Analysis of E-cadherin and WT1 expression in cells cultured on flat and NRA substrata. a. mRNA expression of *CDH1* of cells cultured on flat vs. NRA surfaces. All error bars are S.E.M (n = 3, * = Statistical significance of mRNA expression between cells on flat vs. NRA. $*P<5x10^{-2}$, all two-sided Student's t-test) **b.** Immunoblotting assay of cells cultured on flat surfaces and NRA using WT1 antibody.

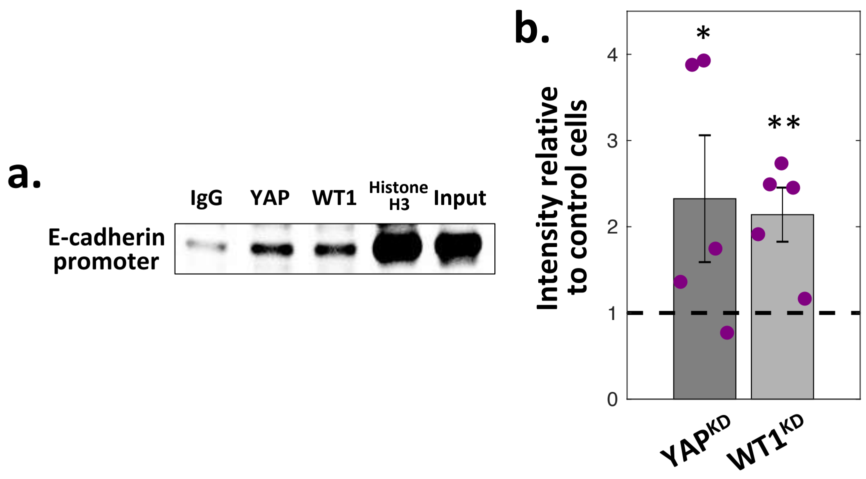

Supplementary Figure 12. Analysis of regulation of E-cadherin transcription by YAP and WT1 a. Chromatin immunoprecipitation using antibodies against YAP, WT1, IgG (negative control) and Histone H3 antibodies (positive control) analyzed by PCR with E-cadherin promoter primer. **b.** Luciferase assay of E-cadherin promoter activity in YAP^{KD} and $WT1^{KD}$ cells compared to wild-type cells. (n = 4, * = statistical significance of the fold changed expression levels of E-cadherin promoters between control and YAP^{KD} (or WT^{KD}) cells. *P<0.05 and **P<0.01, all two-sided Student's t-test).

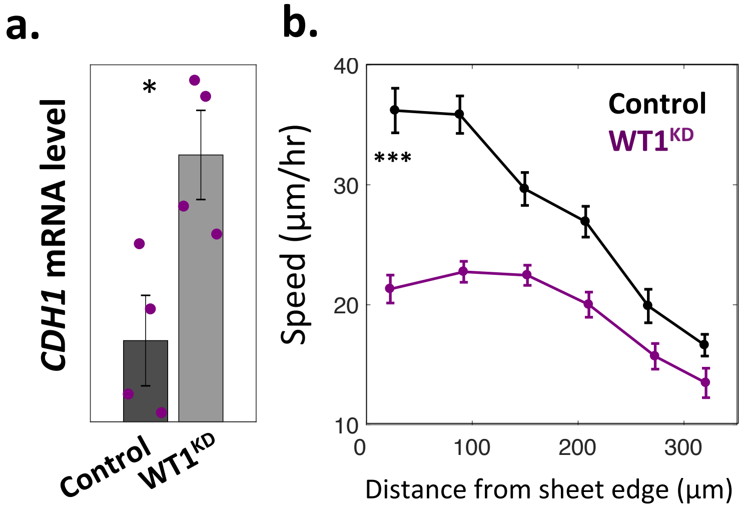

Supplementary Figure 13. Suppressed EMT characteristics in WT^{KD} cells. a. Increased *CDH1* mRNA expression of WT^{KD} cells on NRA. All error bars are S.E.M (n = 3). (* = Statistical significance of mRNA expression between control and $WT1^{KD}$ cells. *P<5x10^{-2}, all two-sided Student's t-test). **b.** Cell migration speed of individual cells in $WT1^{KD}$ epithelial cell sheets as a function of the distance from the sheet edge on NRA pre-coated with fibronectin. All error bars are S.E.M. (* = Statistical significance of control vs. $WT1^{KD}$ cells in the marginal region, ***P<5x10^{-4}, all two-sided Student's t-test.)

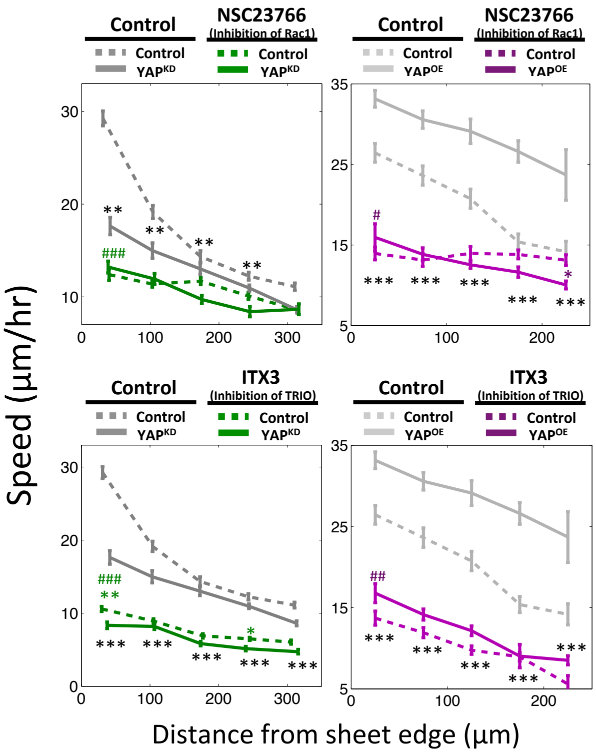

Supplementary Figure 14. Cell migration speed in YAP^{KD} (green, left) and YAP^{OE} epithelial cell sheets (purple, right) as a function of the distance from the sheet edge on NRA in the presence of Rac1 inhibitor, NSC23766 (top) and a TRIO inhibitor, ITX3 (bottom). All error bars are S.E.M. (# = Statistical significance of speed values in the marginal region vs. the most sub-marginal region of YAP^{KD} cells, $^{\#}P<5x10^{-2}$, $^{\#\#}P<5x10^{-4}$, and $^{\#\#\#}P<5x10^{-6}$. * = Statistical significance of control vs. YAP^{KD} cells with drugs (green), control vs. YAP^{OE} cells with drugs (purple) and of YAP^{KD} /YAP^{OE} cells with vs. without drugs (black), *P<0.05, **$P<5x10^{-4}$, and ***$P<5x10^{-6}$, all two-sided Student's t-test.)

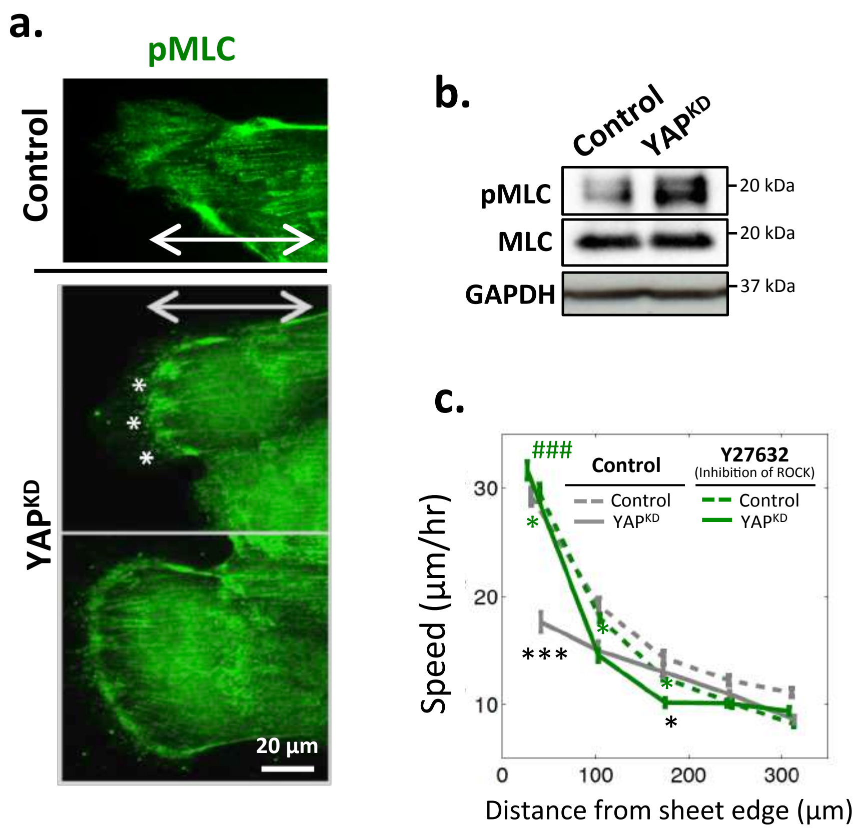

Supplementary Figure 15. YAP regulation of RhoA small GTPase in epithelial cell sheets cultured on NRA. **a.** Immunofluorescence staining of phosphorylated MLC of FLPs in control and YAP^{KD} cells on NRA. The knockdown of YAP partially restores phosphorylated MLC in punctate patterns at the tips of FLPs (marked by *). Double-side arrows indicate the direction of groove/ridge arrays. **b**. Control and YAP^{KD} cells were immunoblotted for evaluating myosin light chain kinase activity using MLC and phosphorylated MLC antibodies **c.** Cell migration speed of individual cells in YAP^{KD} epithelial cell sheets as a function of the distance from sheet edge on NRA with ROCK inhibitor, Y27632. All error bars are S.E.M. (# = Statistical significance of speed on the marginal region vs. the most sub-marginal region of ROCK inhibited YAP^{KD} cells, $^{###}P<5\times10^{-6}$. * = Statistical significance of control vs. YAP^{KD} cells with drugs (green) and of YAP^{KD} cells with vs. without drugs (black), $^{*}P<0.05$, and $^{***}P<5\times10^{-6}$, all two-sided Student's t-test.)

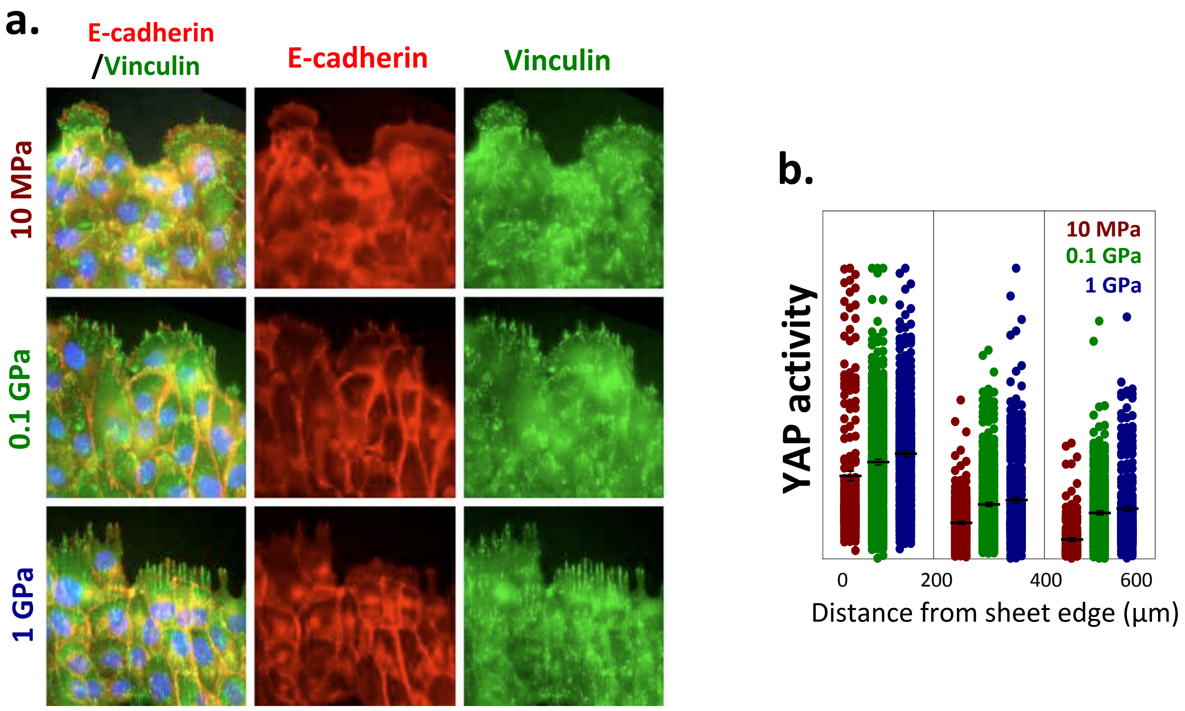

Supplementary Figure 16. Collective cell migration on NRA substrata with different PUA rigidity (10 MPa, 0.1 GPa and 1 GPa) regulated by YAP. **a.** Immunofluorescence staining for E-cadherin and vinculin in epithelial cell sheets cultured on NRA of different rigidity displaying higher aligned/ elongated focal adhesions in FLPs on stiffer NRAs. **b.** Average intensity of YAP staining in nuclei on NRA substrata of different rigidity as a function of the distance from sheet edge. All error bars are S.E.M.

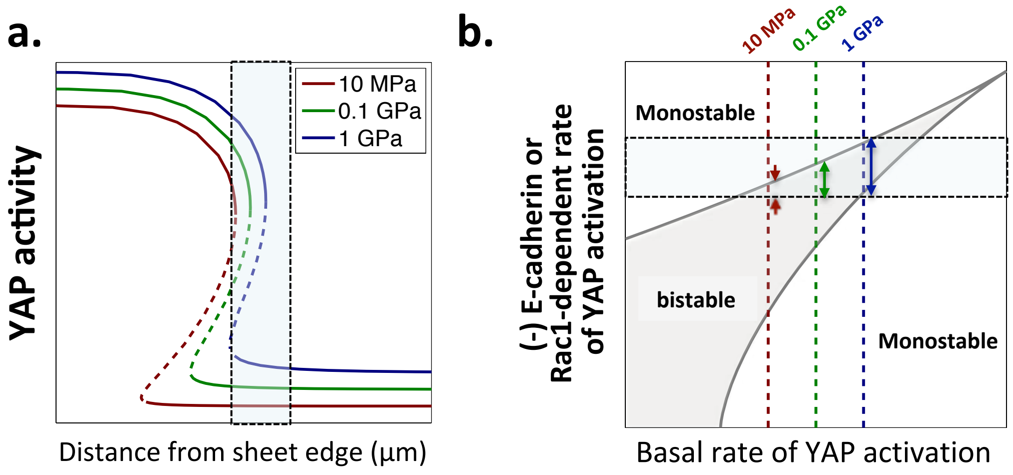

Supplementary Figure 17. Simulation of YAP-regulated signaling networks containing double-negative feedback with E-cadherin or positive feedback with Rac1. a. Simulated YAP activity as a function of distance from sheet edge for different assumed rigidity values, represented by different basal activation of YAP (see the model description in the Supplementary discussion). A shaded region corresponds to the regions of epithelial sheets where we experimentally observed nuclear localization of YAP. **b.** Two-parameter bifurcation diagram relating E-cadherin- or Rac1-dependent rates of YAP activation and basal rate of YAP activation for different NRA rigidity values. This diagram shows the range of parameters where the system has bi-stability. Dashed lines indicate basal rates of YAP activation corresponding to assumed substrate rigidity values of NRA. Double-sided arrows indicate the range of E-cadherin- or Rac1- dependent rate of YAP activation where the output exhibits bi-stability for the specific rigidity values.

Figure 1e

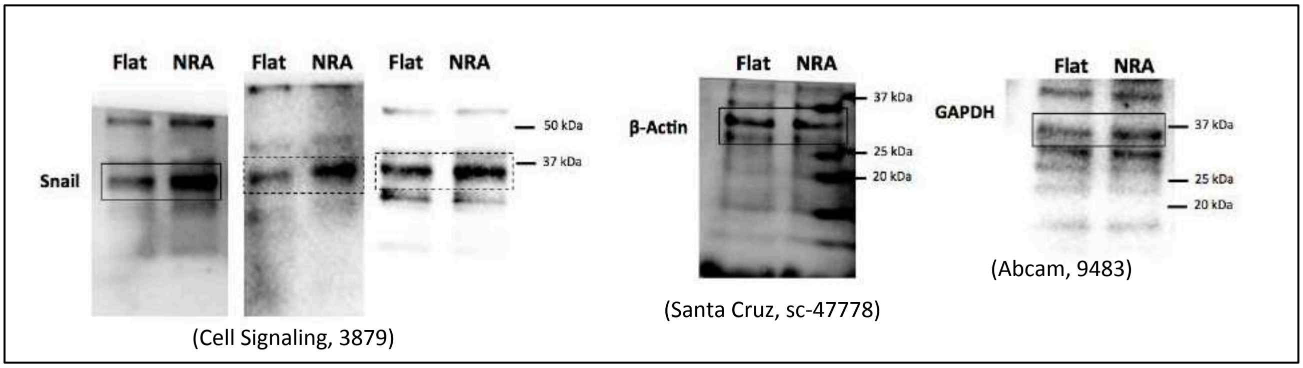

Figure 2a

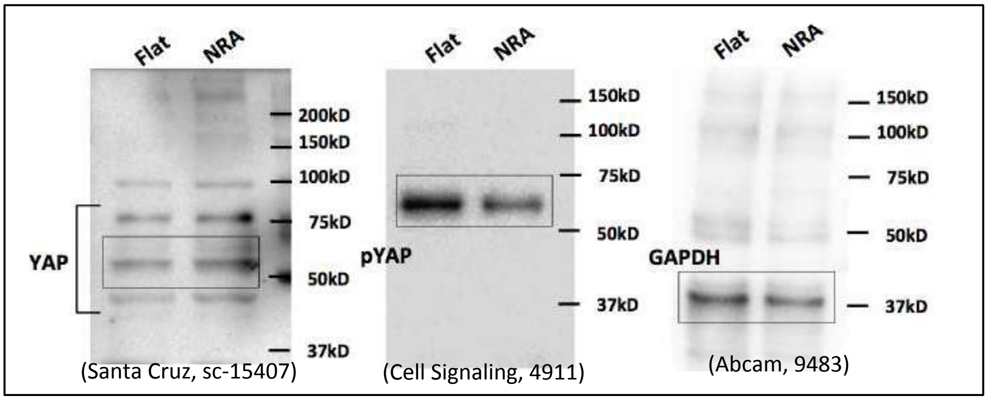

Figure 2g

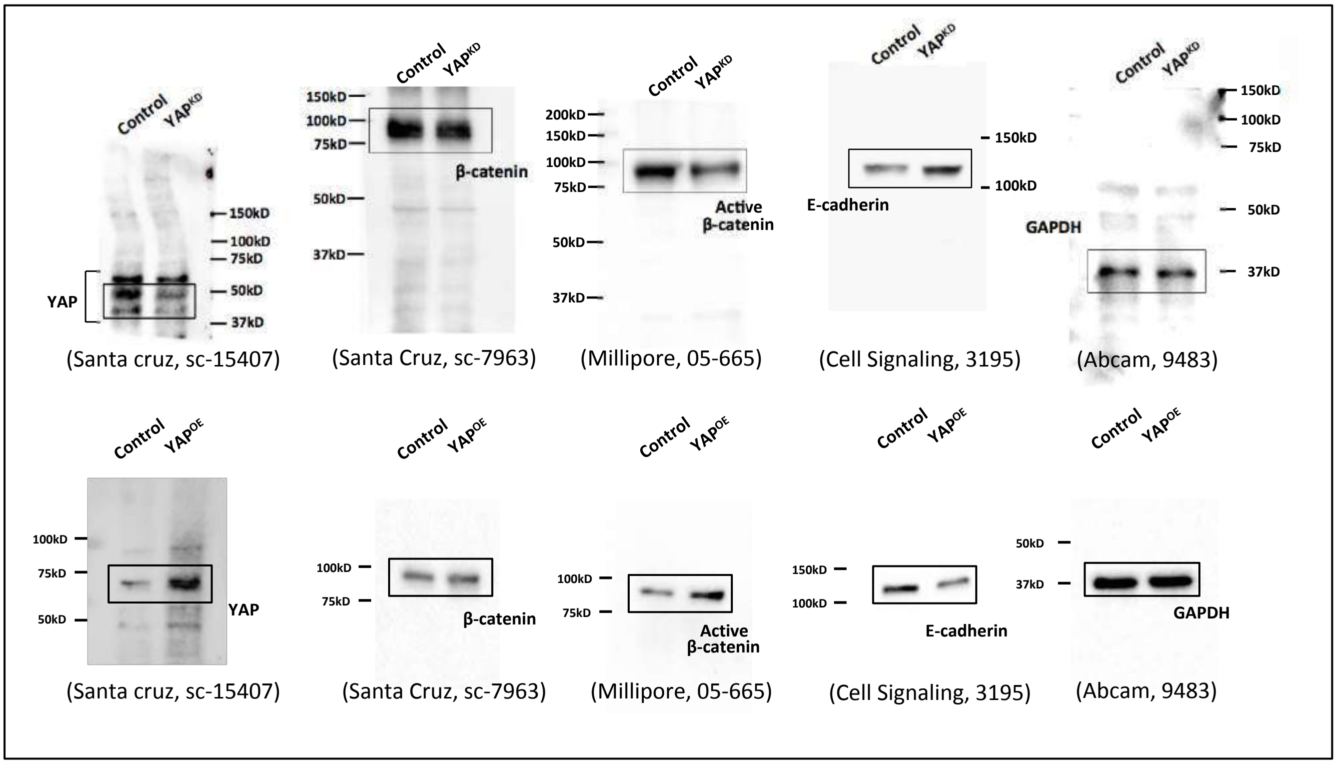

Supplementary Figure 18 (Continued). Raw images of immunoblotting experiments

Figure 3b

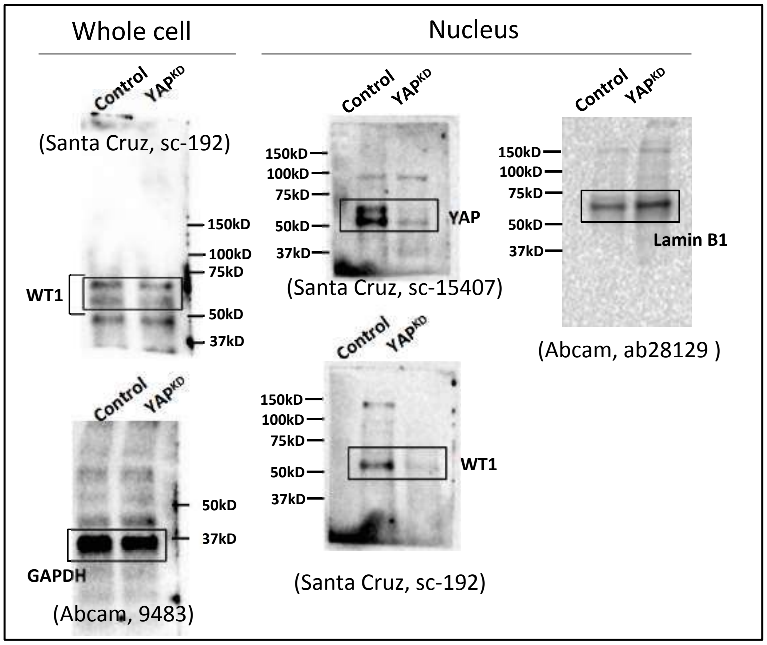

Figure 3d

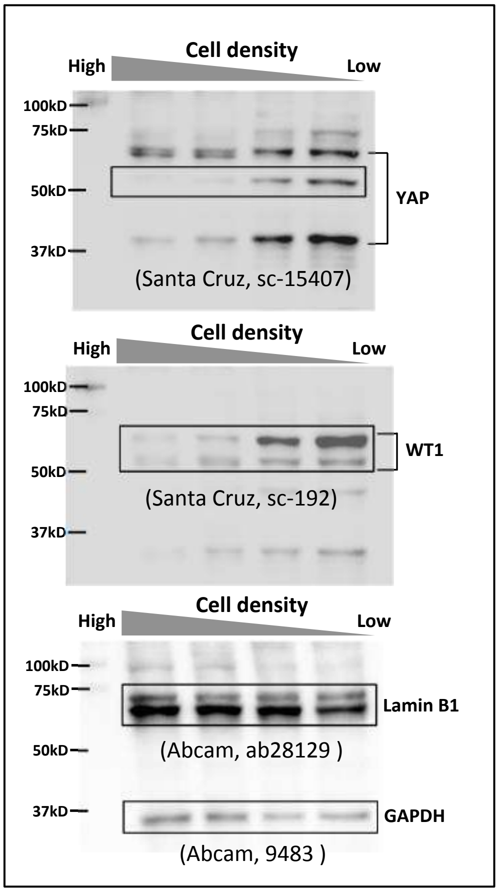

Figure 4b

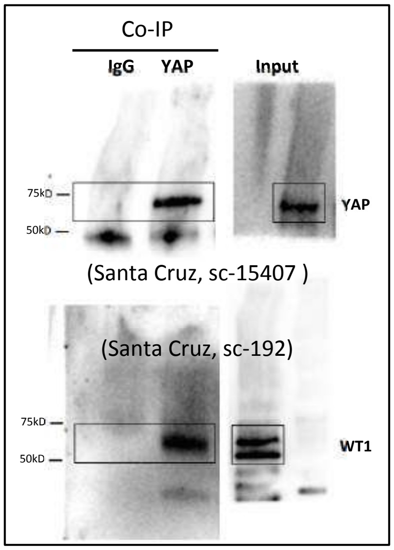

Figure 4d

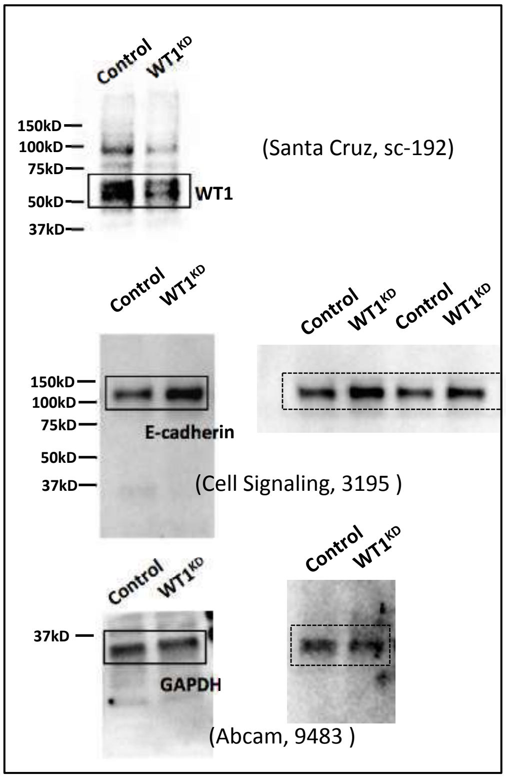

Figure 4g

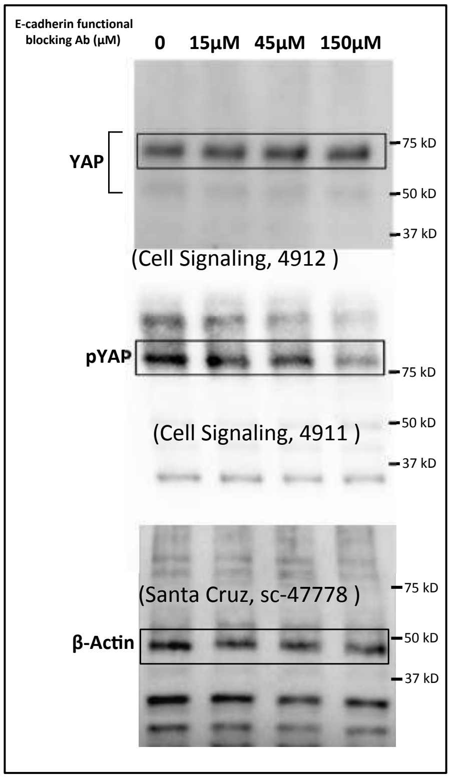

Supplementary Figure 18 (Continued). Raw images of immunoblotting experiments

Figure 5a

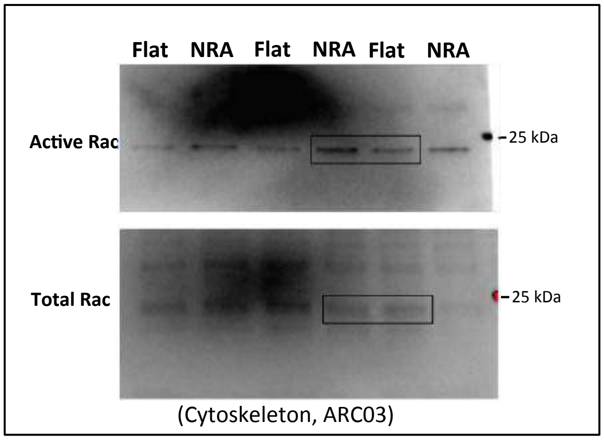

Figure 5b

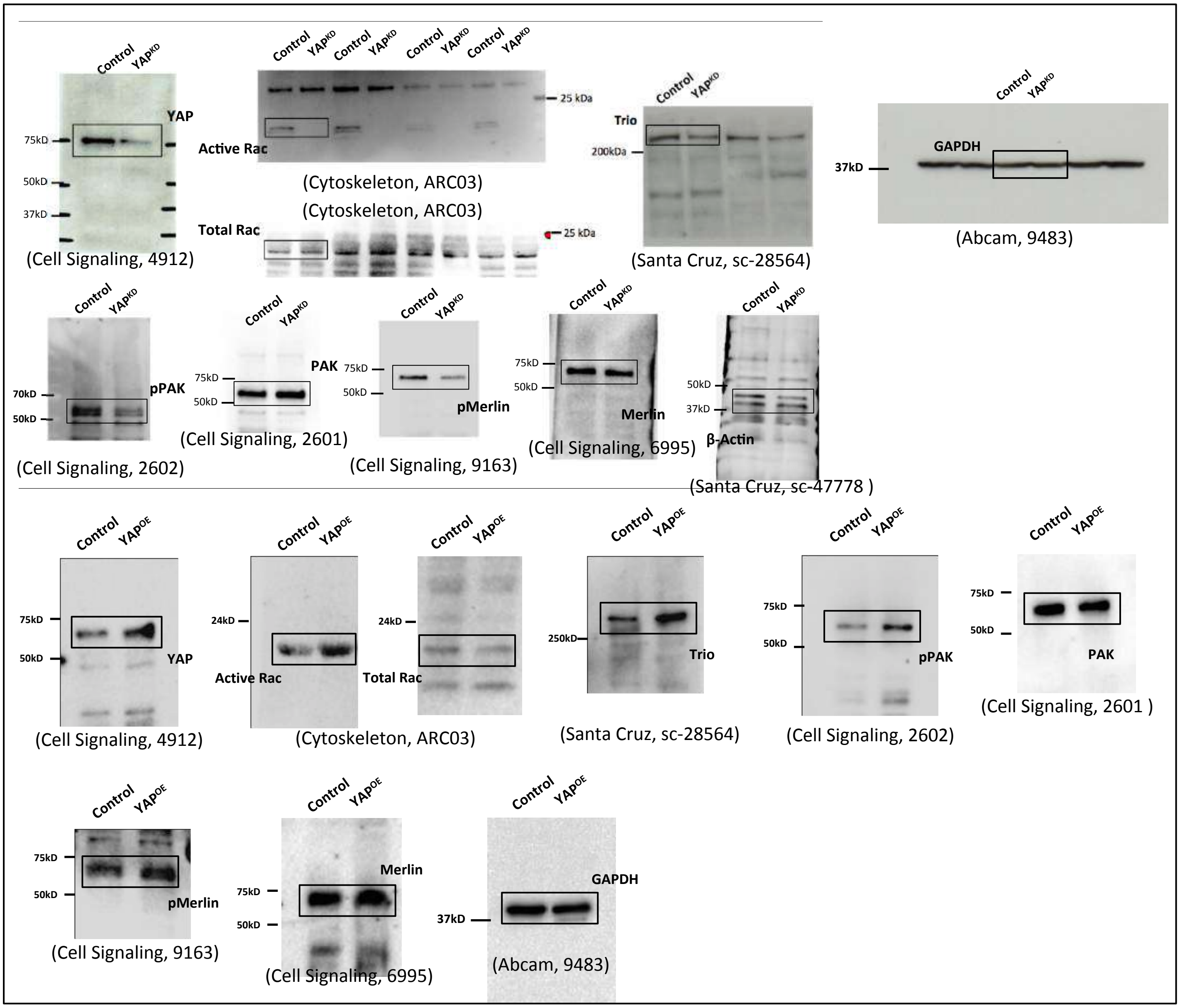

Supplementary Figure 18 (Continued). Raw images of immunoblotting experiments

Figure 6b

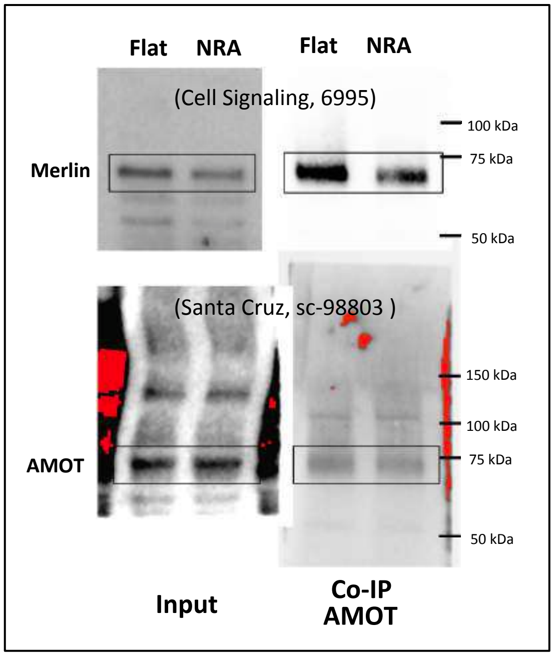

Figure 6c

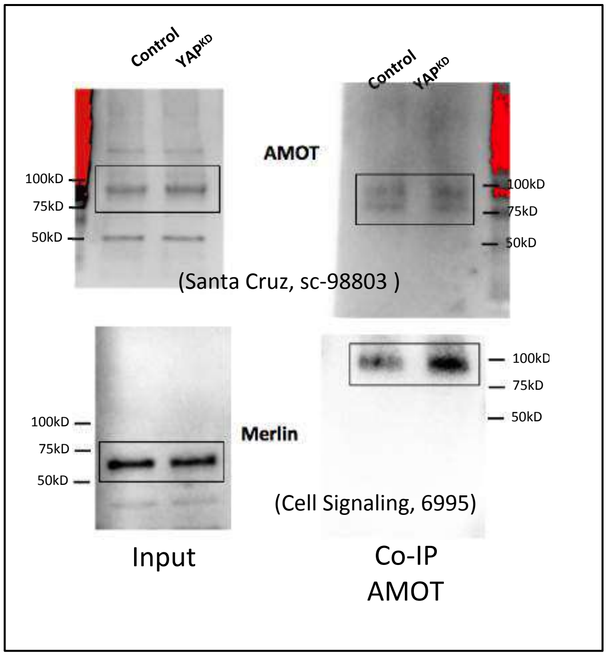

Figure 6d

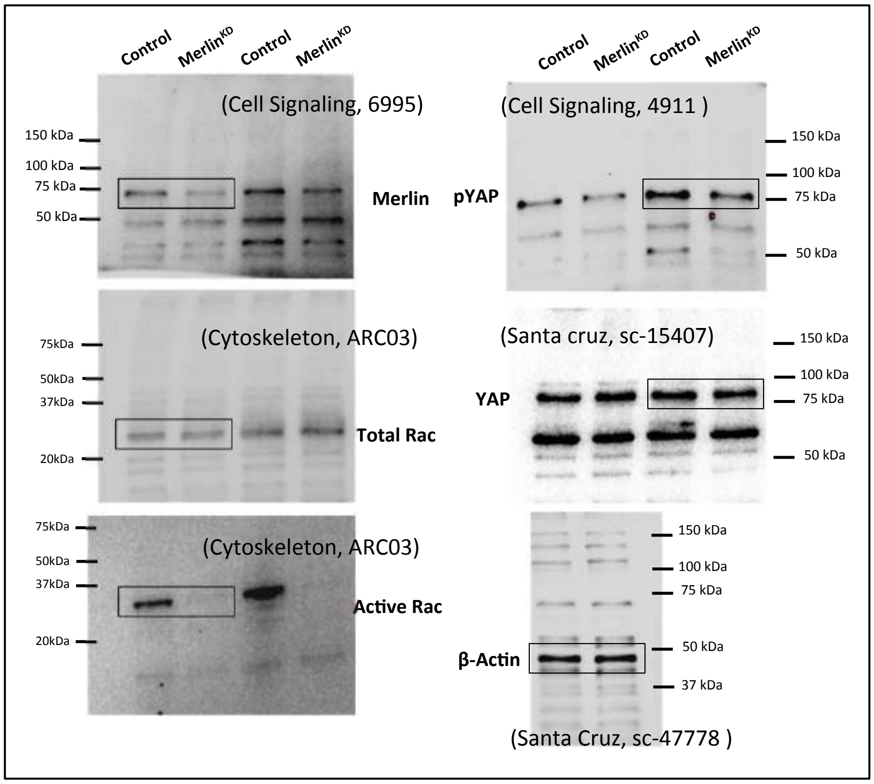

Supplementary Figure 18 (Continued). Raw images of immunoblotting experiments

Figure 8a

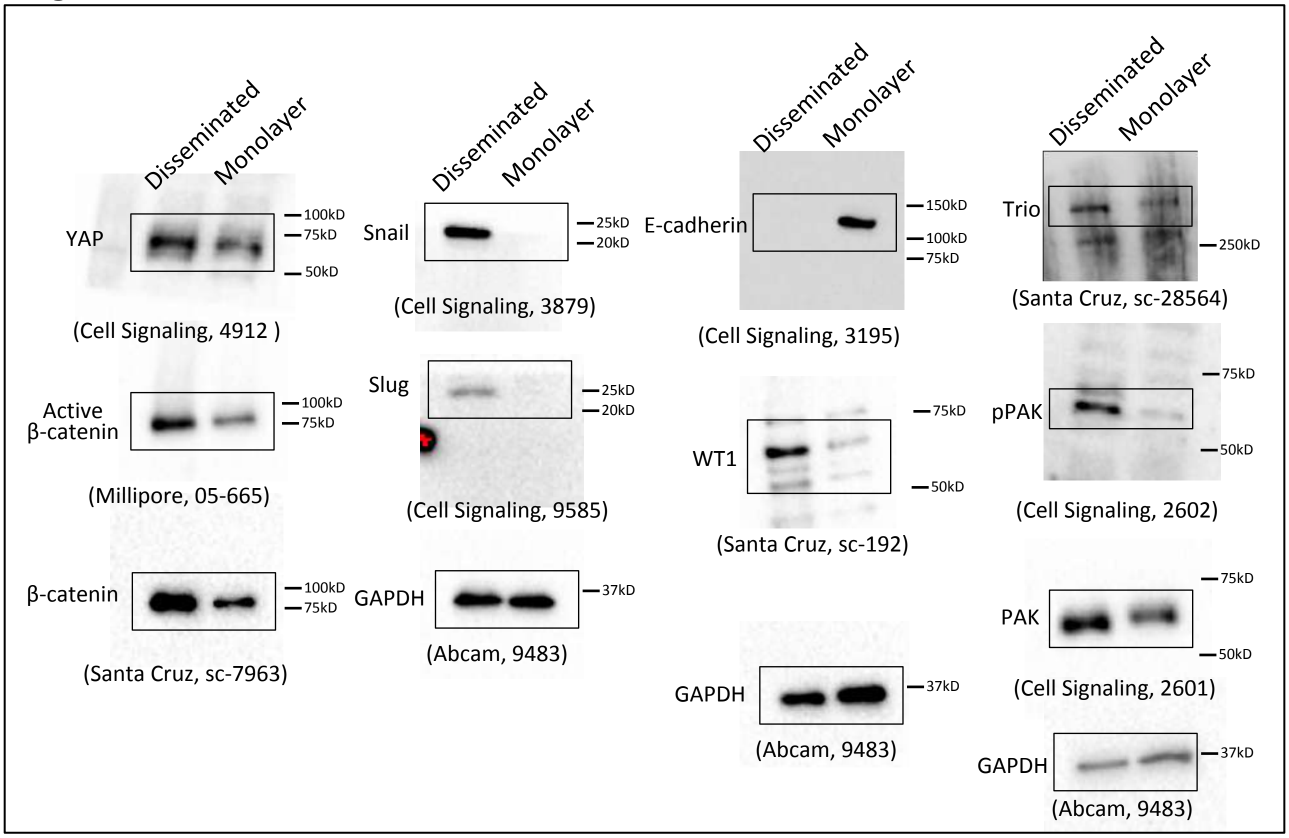

Supplemental figure 11b

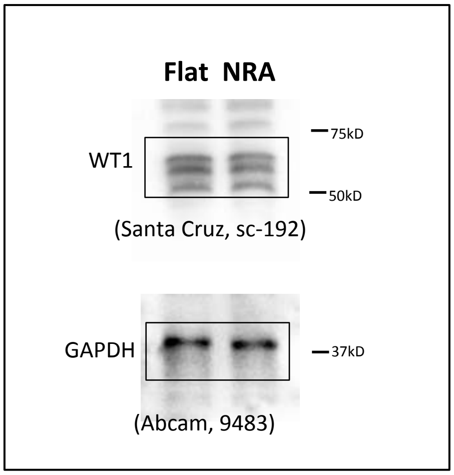

Supplemental figure 15b

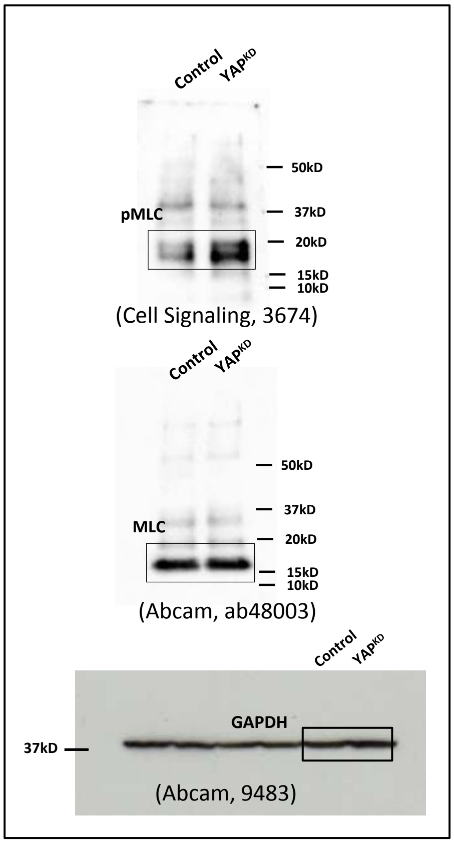

Supplementary Figure 18. Raw images of immunoblotting experiments

Supplementary Discussion

1. Computational modeling of YAP driven signaling network regulating EMT

The YAP-driven signaling networks determining EMT and cell dissemination from epithelial sheets on NRA were modeled as a combination of reactions suppressing cell-cell adhesion through down-regulation of E-cadherin complexes and enhancing individual cell migration via Rho-family GTPases, particularly Rac1. The inhibitory effects of E-cadherin and stimulatory effect of Rac1 on YAP activation identified in our study suggest feedback interactions. The corresponding signaling processes summarized in Fig. 4h and Fig. 6g were modeled as a series of ordinary differential equations below. As many details of these processes remain to be elucidated, the model equations are simplified versions of the interactions capturing the feedback character:

$$\frac{d}{dt}[\mathrm{YAP}_{\text{E-cadherin}}] = k_{\mathrm{YAP}} - k_{\mathrm{YAP\cdot E\text{-}cadherin}}[\text{E-cadherin}] - d_{\mathrm{YAP}}[\mathrm{YAP}_{\text{E-cadherin}}]$$

$$\frac{d}{dt}[\text{E-cadherin}]$$

$$= C_{\text{E-cadherin}} - k_{\text{E-cadherin}} \frac{[\mathrm{YAP}_{\text{E-cadherin}}]^3}{K_{\text{E-cadherin}}^3 + [\mathrm{YAP}_{\text{E-cadherin}}]^3} - d_{\text{E-cadherin}}[\text{E-cadherin}]$$

(Fig. 4h)

$$\frac{d}{dt}[\mathrm{YAP}_{\mathrm{Rac1}}] = k_{\mathrm{YAP}} + k_{\mathrm{YAP\cdot Rac1}}[\mathrm{Rac1}] - d_{\mathrm{YAP}}[\mathrm{YAP}_{\mathrm{Rac1}}]$$

$$\frac{d}{dt}[\mathrm{Rac1}] = k_{\mathrm{Rac1}} \frac{K_{\mathrm{Rac1}}^3[\mathrm{YAP}_{\mathrm{Rac1}}]^3}{K_{\mathrm{Rac1}}^3 + [\mathrm{YAP}_{\mathrm{Rac1}}]^3} - d_{\mathrm{Rac1}}[\mathrm{Rac1}] - D_{\mathrm{Rac1}}$$

(Fig. 6g)

A major assumption underlying the model is that the dependence of E-cadherin and Rac1 on YAP activity are non-linear and saturable. These assumptions are necessary to obtain switch-like, bi-stable behavior. At the steady state, all the time derivatives are equal to zero, leading to the following algebraic equations:

$$0 = k_{\mathrm{YAP}} - k_{\mathrm{YAP\cdot E\text{-}cadherin}}[\text{E-cadherin}] - d_{\mathrm{YAP}}[\mathrm{YAP}_{\text{E-cadherin}}]$$

$$0 = C_{\text{E-cadherin}} - k_{\text{E-cadherin}} \frac{[\mathrm{YAP}_{\text{E-cadherin}}]^3}{K_{\text{E-cadherin}}^3 + [\mathrm{YAP}_{\text{E-cadherin}}]^3} - d_{\text{E-cadherin}}[\text{E-cadherin}]$$

(Fig. 4h)

$$0 = k_{\text{YAP}} + k_{\text{YAP}\cdot\text{Rac1}}[\text{Rac1}] - d_{\text{YAP}}[\text{YAP}_{\text{Rac1}}]$$

$$0 = k_{\text{Rac1}} \frac{K_{\text{Rac1}}^3[\text{YAP}_{\text{Rac1}}]^3}{K_{\text{Rac1}}^3 + [\text{YAP}_{\text{Rac1}}]^3} - d_{\text{Rac1}}[\text{Rac1}] - D_{\text{Rac1}}$$

(Fig. 6g)

These equations with parameters and their assumed values, listed in Supplementary Table 1, constitute a full description of YAP-driven signaling network. In particular, we note the value of $k_{\text{YAP-E-cadherin}}$ represents a negative effect of E-cadherin on YAP in the double negative feedback determining cell-cell adhesion, $k_{\text{E-cadherin}}$ represents negative regulation of YAP on E-cadherin resulting from binding of YAP-WT1 transcription complex to the E-cadherin promoter. Similarly, we take $k_{\text{YAP-Rac1}}$, which indicates a positive effect of Rac1 on YAP, whereas the k_{Rac1} accounts for the corresponding positive effect of YAP on Rac1 through facilitation of dissociation of Merlin from AMOT in the positive feedback regulating cell migration speed. The parameters are arbitrary, but the results are meant to be semi-quantitative in nature, to demonstrate the effect of the feedback interaction. Overall, the outcome of the model is qualitatively robust to these values, as demonstrated by the bifurcation diagram in Supplementary Fig. 17.

Each pair of equations generates two 'null clines' whose intersections are the steady states of the system. Given the model non-linearity, three steady states are robustly obtained, one unstable and two stable ones. This is the common 'bi-stability' regime, suggesting two mutually exclusive states in a switch-like regulation: if E-cadherin is low and Rac1 is high, YAP is high. Conversely, if Rac1 is low and E-cadherin is high, YAP is low (Fig. 7a). We suggest that these two states represent a mesenchymal state (low E-cadherin, high Rac1 and high YAP) and epithelial state (high E-cadherin, low Rac1 and low YAP) in epithelial-mesenchymal transition. This is also consistent with the bimodal distribution of EMT markers such as nuclear translocation of active β-catenin and YAP, as a function of the distance from sheet edge, as shown in Figs 2c and 2d. This behavior is robust to changes within the range of the parameter values, E-cadherin- or Rac1-dependent rates of YAP activation and the basal rate of YAP activation as long as the null clines continue to intersect in 3 distinct points, preserving the bi-

stable nature of the output (see the bifurcation analysis in Supplementary Fig. 17). This model predicts that the range of where the system has bimodal distribution of EMT markers becomes wider as the basal rate of YAP activation increases. This is an important model prediction, validated in Fig. 7 of the main text. In this figure, we observed the extension of bi-stable region with increasing substratum rigidity, the input that we assume to correspond to higher values of basal rate of YAP activation (Fig. 7c and Supplementary Fig. 17). Basal rates of YAP activation for each NRA rigidity value are listed in Supplementary Table 2.

Supplementary Table 1

Parameter	unit	Value	Description
k_{YAP}	μM s^{-1}	1	Basal rate of YAP activation
$k_{\mathrm{YAP\text{-}E\text{-}cadherin}}$	s^{-1}	1.8	E-cadherin-dependent rate of YAP activation
$k_{\mathrm{YAP\text{-}Rac1}}$	s^{-1}	1.8	Rac1-dependent rate of YAP activation
$k_{\mathrm{E\text{-}cadherin}}$	s^{-1}	0.9	YAP-dependent rate of E-cadherin expression
k_{Rac1}	s^{-1}	0.9	YAP-dependent rate of Rac1 activation
$K_{\mathrm{E\text{-}cadherin}}$	μM	1	Dissociation constant of YAP-WT1 transcriptional constant
K_{Rac1}	μM	1	Michaelis-like constant of Rac activation
d_{YAP}	s^{-1}	1	Inactivation rate of YAP
$d_{\mathrm{E\text{-}cadherin}}$	s^{-1}	1	Degradation rate of E-cadherin
d_{Rac1}	s^{-1}	1	Inactivation rate of Rac1
$C_{\mathrm{E\text{-}cadherin}}$	s^{-1}	0.9	Initial activation rate of E-cadherin
D_{Rac1}	s^{-1}	0.9	Degradation constant of Rac1

Supplementary Table 2

Parameter	unit	Value	Description
$k_{\mathrm{YAP,\ 10\ MPa}}$	μM s^{-1}	0.95	Basal rate of YAP activation on 10 MPa NRA
$k_{\mathrm{YAP,\ 0.1\ GPa}}$	μM s^{-1}	1	Basal rate of YAP activation on 0.1 GPa NRA
$k_{\mathrm{YAP,\ 1\ GPa}}$	μM s^{-1}	1.05	Basal rate of YAP activation on 1 GPa NRA
