# Supplementary material for: Switch-like enhancement of epithelial-mesenchymal transition by YAP through feedback regulation of WT1 and Rho-family GTPases
Source: Nat Commun. 2019 Jun 26;10:2797. doi: 10.1038/s41467-019-10729-5 (PMC6594963; doi:10.1038/s41467-019-10729-5)
Supplement: Supplementary file 2 — Description of Additional Supplementary Files [file 41467_2019_10729_MOESM2_ESM.pdf]

Description of Additional Supplementary Files

File Name: Supplementary Movie 1
Description: Collective migration of cells in an epithelial cell sheet on a flat substratum and NRA

File Name: Supplementary Movie 2
Description: Actin of marginal and sub-marginal cells in an epithelial cell sheet on NRA

File Name: Supplementary Movie 3
Description: α-microtubule of cells at the tip and convex region in the boundary of an epithelial cell sheet on NRA

File Name: Supplementary Movie 4
Description: Dissemination of 'tip' cells on NRA with EMT inducer, TGFβ

File Name: Supplementary Movie 5
Description: Collective migration of control and YAPKD cells on NRA

File Name: Supplementary Movie 6
Description: Collective migration of control and YAPKD cells on NRA with E-cadherin inhibition

File Name: Supplementary Movie 7
Description: Collective migration of control and WT1KD cells on NRA the presence of the E-cadherin blocking antibody

File Name: Supplementary Movie 8
Description: Collective migration of control and YAPKD cells on NRA with Rac1 and TRIO inhibition

File Name: Supplementary Movie 9
Description: Collective migration of control and YAPOE cells on NRA with Rac1 and TRIO inhibition

File Name: Supplementary Movie 10
Description: Collective migration of control and MerlinKD cells on NRA with Rac1 inhibition

File Name: Supplementary Movie 11
Description: Collective migration of control and YAPKD cells on NRA with ROCK inhibition
